# Optimizing the Au Particle Doping Size for Enhanced Photocatalytic Disinfection under Low-Intensity Visible Light

**DOI:** 10.1021/acsnano.5c07650

**Published:** 2025-07-24

**Authors:** Gi Byoung Hwang, Ki Joon Heo, Woongkyu Jee, Luca Panariello, Jacopo Piovesan, Mabel Cornwell, Alberto Collauto, Andreas Kafizas, Shanom Ali, Caroline Knapp, Alexander J. MacRobert, Asterios Gavriilidis, Ivan P. Parkin, Scott M. Woodley, Jae Hee Jung

**Affiliations:** † Department of Chemistry, 4919 University College London, 20 Gordon Street, London WC1H 0AJ, U.K.; ‡ School of Mechanical Engineering, Chonnam National University, 77 Yongbong-ro, Gwangju 61186, Republic of Korea; ¶ Department of Chemical Engineering, 4919 University College London, Torrington Place, London WC1E 7JE, U.K.; ∥ Department of Chemistry, 4615 Imperial College London, Molecular Science Research Hub, White City Campus, 82 Wood Lane, London W12 OBZ, U.K.; ⊥ Environmental Research Laboratory, University College London Hospitals NHS Foundation Trust, 235 Euston Road, London NW1 2BU, U.K.; # Division of Surgery and Interventional Science, UCL, Royal Free Campus, Rowland Hill Street, London NW3 2PF, U.K.; ∇ Department of Mechanical Engineering, 35006 Sejong University, 209 Neungdong-ro, Seoul 05006, Republic of Korea

**Keywords:** gold nanoparticles, photocatalyst, disinfection, bacteria, gold nanocluster, charge carrier, photoexcitation

## Abstract

Here, we present the effect of 1.2–9.9 nm Au particles on crystal violet-treated polymer under a low intensity of visible light. The use of Au particles ≤ 6.3 nm promoted charge carrier transfer from crystal violet to Au particles. Photospectroscopy analyses and DFT computations revealed that a change in the electronic band structure caused by the size reduction of the particle altered the charge carrier transfer pathway in crystal violet. Especially for crystal violet—1.2 nm Au particles, charge carrier transfer predominantly occurs at the S_1_ of crystal violet because the T_1_ state lacks sufficient potential energy for transfer. 1.2 nm Au particles on crystal violet not only most significantly enhanced the generation of O_2_•^–^, H_2_O_2_, and ^•^OH by minimizing unnecessary side reactions or energy loss but also showed the most potent disinfection activity against , even at low visible light flux levels (0.037–0.054 mW cm^–2^), which resulted in a 5.3 log reduction in viable bacteria after 6 h exposure to visible light. This finding provides fundamental insights into the Au effect as a cocatalyst in photocatalysts and the development of light-activated self-sterilizing surfaces that can be applied to various hospital surfaces to prevent the spread of pathogens, which remains a global challenge.

## Graphical abstract

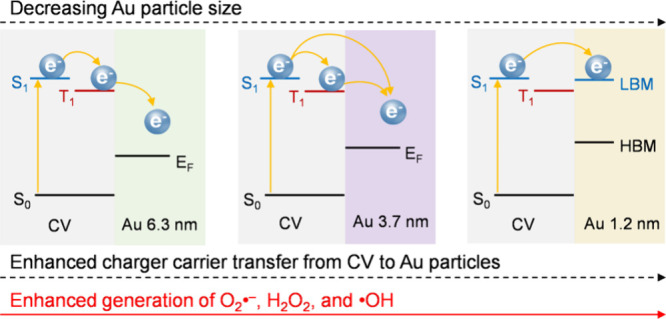

Biofilm poses one of the most significant challenges to 21st century medical care,^1^ particularly concerning healthcare-associated infections (HAIs), which are infections that patients acquire during medical or surgical treatments or through contact with healthcare workers.^2^ Globally, HAIs impact >100 million hospitalised patients annually.^3,4^ Surface contamination by pathogens is responsible for > 80% of microbial infections and > 60% of all HAI cases.^3,4^ These infections, caused by a variety of microorganisms, lead to a range of outcomes, ranging from patient discomfort to permanent disability and, in severe cases, even death.^5^ Many of these exhibit resistance to antibiotic treatments, necessitating surgical intervention for effective treatment.^4,6^ The global cost of managing biofilm-associated infections is estimated to be nearly $300 billion annually.^7^

Vigorous surface cleaning or disinfection schemes have been actively implemented in hospitals to prevent surface contamination, which can serve as a reservoir for pathogens contributing to the spread of HAIs.^5,8^ However, the conventional protocols have been reported to be inefficient in preventing surface contaminations:^8^ for instance, previous studies showed that 60% of surfaces of hospital rooms remained contaminated by complex (ABC) after terminal cleaning and disinfection, and 26.6% of surfaces were still contaminated by either ABC or methicillin-resistant after four rounds of terminal cleaning and disinfection, indicating the critical need for efficient strategies.^9–11^

Visible-light-active disinfection surfaces have gained significant attention for their potential to prevent HAI incidents and to maintain sterile surfaces.^12–19^ By utilizing visible light, which constitutes a substantial portion of indoor light,^16,20^ these surfaces can generate reactive oxygen species (ROS) lethal to pathogens.^14,15,17,20,21^ They are effective not only in disinfecting antibiotic-resistant pathogens but also in degrading organic contaminants.^22,23^ Various strategies, including organic photocatalysts, heterojunction architectures, metal nanoparticles, and nitrogen doping, have been employed to develop light-activated disinfection surfaces.^24–26^ Among various strategies, doping photocatalysts with metal particles is one of the most extensively studied approaches for achieving visible light activation.^14,27^ There are two strategies for incorporating metal particles into photocatalysts. First, metal particles exhibiting plasmonic behavior under visible light can be loaded onto a UV-active photocatalyst. This enhances photocatalytic reactions through hot-electron transfer, near-field enhancement, and resonance energy transfer,^28–32^ leading to enhanced disinfection activity.^26,33^ Second, metal particles can be used in combination with a visible-light active photocatalyst, where the metal acts as an electron acceptor from photoexcited dye molecules,^34–36^ facilitating ROS generation;^34,35,37,38^ while this approach demonstrates promising photocatalytic disinfection performance,^37,38^ the underlying mechanisms, particularly the interactions between the photocatalyst and metal particles, are relatively less explored compared to those driven by plasmonic enhancement.

Despite numerous studies on visible-light-active disinfection surfaces, their real-world application to healthcare facilities and other indoor settings is challenging, as most studies require intense visible light to achieve adequate pathogen kill. For instance, Au/PCN-224/Cu­(II)-modified cotton fabrics achieved complete inactivation of and under xenon lamp irradiation at 300 mW cm^–2^ (λ ≥ 420 nm).^33^ Similarly, an Ag-decorated TiO_2_ surface demonstrated potent bactericidal effects under visible light but required an intensity of 30 mW cm^–2,^ ^39^ which is significantly higher than typical indoor lighting levels (∼0.05 mW cm^–2^). As an alternative strategy, combining dyes with metal particles has emerged as a promising approach to achieve antibacterial effects under low-intensity visible light. Crystal violet and ZnO particle-embedded silicone surfaces effectively killed and under white hospital lighting at 0.6 mW cm^–2^,^37^ and similar results were obtained in an Au particle-dye hybrid system against and at 1.0 mW cm^–2^ of white light.^38^ However, the fundamental mechanisms, particularly the role of metal particles in charge carrier dynamics, remain poorly understood. The particle size is identified as a critical factor in the improvement of photocatalytic reactions. Although there is an argument about whether smaller or larger particle sizes are more beneficial for enhancing photocatalytic reactions, it is widely acknowledged that optimizing particle size can significantly improve reaction efficiency.^40–43^

In the present study, we have explored the optimal size of Au particles, ranging from clusters to nano regions, to efficiently enhance the photodisinfection activity of crystal violet-treated polymer under low visible light flux levels. Photospectroscopy analyses and density functional theory (DFT) computations showed that doping Au particles ≤ 6.3 nm on crystal violet-treated polymer promoted the movement of photoexcited charge carriers from the organic photocatalyst to Au particles, and the charge carrier behavior was varied depending on the size of Au particles on the photocatalyst. This enhanced charge carrier movement resulted in an increased generation of ROS, including superoxide radical (O_2_•^–^), hydrogen peroxide (H_2_O_2_), and hydroxyl radical (^•^OH) in a size-dependent manner, whereas singlet oxygen (^1^O_2_) generation decreased. Testing these materials against showed that the combination of crystal violet and 1.2 nm Au particles in the polymer has very potent photobactericidal activity, even at a very low intensity of visible light.

## Results and Discussion

Figure 1a,b shows the shapes and size distributions of synthesized Au particles over nano- and cluster-size regions. For the synthesis of Au particles ranging from 3.7 to 9.9 nm in size, a seeded-growth method of citrate-capped metal nanoparticles was employed, while cysteine-capped Au_25_ clusters were synthesized using carbon monoxide as a reducing agent for Au particles of 1.2 nm in size.^44,45^ High resolution transmission electron microscope (HRTEM) analysis showed that most synthesized Au particles are spherical (Figure 1(a-1 to 4)). However, the shape of the smallest Au particles was not clearly discernible due to their tiny size (inset in Figure 1(a-5)). The size distributions for Au particles in the nanoregion were 3.7 ± 0.4, 6.3 ± 0.6, 8.6 ± 0.8, and 9.9 ± 0.6 nm, respectively (Figure 1b), with their size deviation being ≤10% at each condition. The cluster’s size was 1.2 ± 0.1 nm, comparable to the Au_25_ cluster’s size reported by previous studies.^46^ It was confirmed that the differences in particle sizes under five different synthesis conditions are statistically significant (One-way analysis of covariance (ANOVA): *P*-value <0.01). As shown in Supporting Figure 1, the particles were well dispersed without any agglomeration. To identify the composition of the 1.2 nm particles, electrospray ionization mass spectrometry (ESI-MS) was employed. As shown in Figure 1c, it was observed that the ESI-MS spectrum for 1.2 nm Au particles had the most intense peak at *m*/*z* 2361.45 (#1) in a range of *m*/*z* 1500–4000 along with similar small peaks (#2–7) matching to H^+^ dissociation and Na^+^ coordination to [Au_25_(Cys)_18_] gold cluster. Isotope pattern analysis of peaks #1–7 shows that [Au_25_(Cys)_18_] carries three negative charges, and their ionized species and isotope pattern peaks match well those obtained by theoretical simulation of [Au_25_(Cys)_18_] (Supporting Note 1). UV–vis absorbance spectroscopy shows that the absorbance spectrum of 1.2 nm Au particles differed from that of the larger Au particles. 1.2 nm Au particles gave multiple absorption peaks at 400, 450, 670, and 780 nm, while the particles ≥3.7 nm in size gave a main absorption at 510 nm, and the absorption intensity increased with the size of Au particles (Figure 1d).

**Figure 1:**
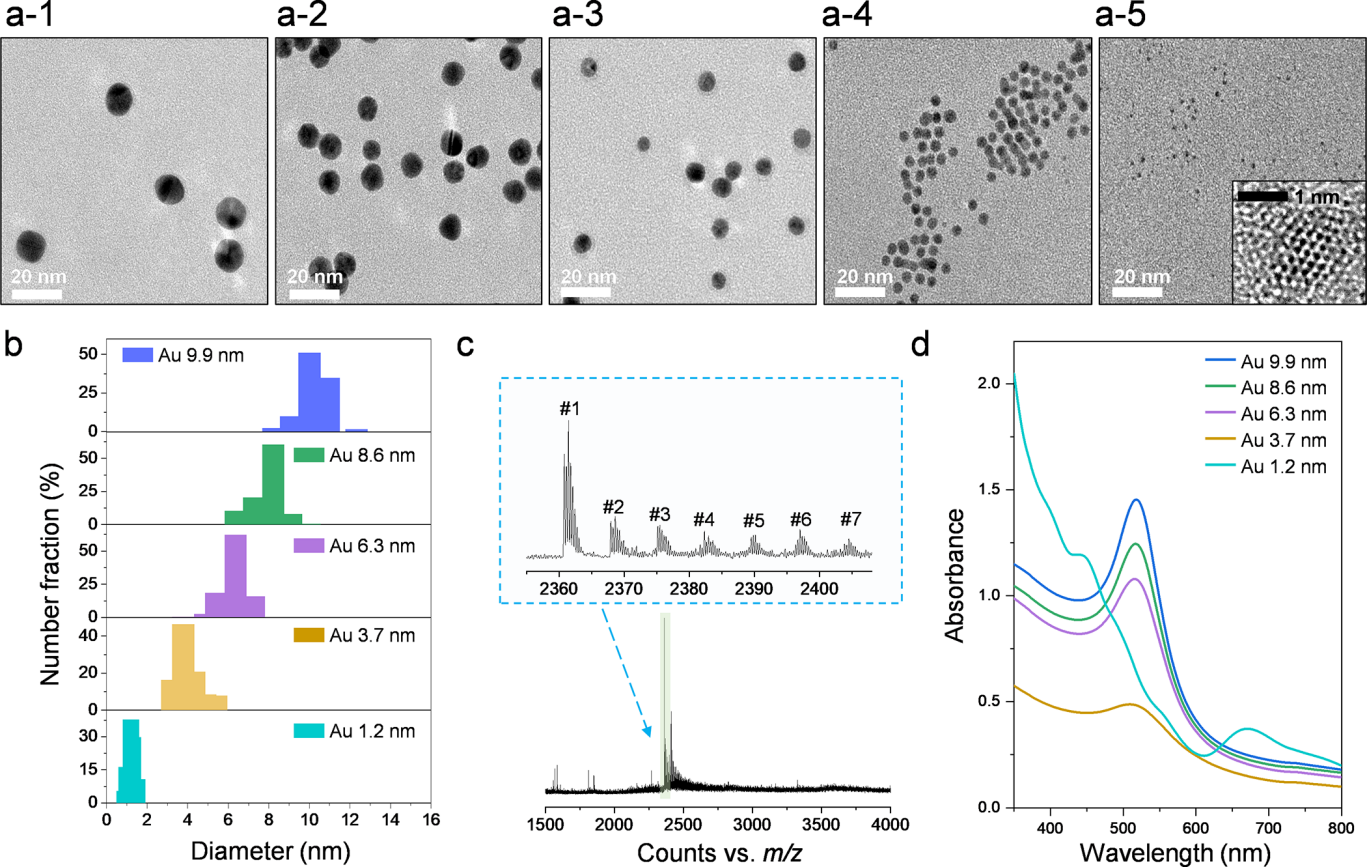
(a) High resolution transmission electron microscopy (HRTEM) images of Au particles ranging from 1.2 to 9.9 nm in size. a-1, Au 9.9 nm. a-2, Au 8.6 nm. a-3, Au 6.3 nm. a-4, Au 3.7 nm. a-5, Au 1.2 nm (inset: magnified view). (b) Size distribution of the Au particles. (c) Electrospray ionization-mass spectrum (ESI-MS) of 1.2 nm Au particles, which are cysteine-capped Au_25_ clusters ([Au_25_(Cys)_18_]). 3.7–9.9 nm Au particles are citrate-capped nanoparticles. (d) UV–vis absorbance spectra of Au particles in wavelengths of 350–800 nm.

Crystal violet and Au particles were coated onto polymer surfaces using an encapsulation process.^37^ As shown in Supporting Figure 3, the surface color of the crystal violet-treated polymer was violet, and the addition of Au particles to the treated surface did not affect the surface color. All crystal violet-treated polymers exhibited a main absorbance peak at ∼600 nm, with a shoulder peak at 530 nm (Figure 2a). However, contrary to other treated polymers, a significant absorbance increase at 530 nm was observed on the crystal violet-treated polymer with 1.2 nm Au particles. This enhancement is attributed to changes in the aggregation state or orientation of crystal violet molecules, likely induced by interactions between the 1.2 nm Au particles and the photocatalyst. According to previous studies, such interaction could alter the π-orbital overlap between adjacent cationic dye molecules, resulting in an increase in absorbance or a blue shift.^47–49^ As shown in Supporting Figure 4a, in the absence of crystal violet, the absorbance peak characteristic of Au particles was not detected in any of the tested polymer samples. This may be due to the strong background absorption of the silicone polymer, which could mask the distinct absorption features of the Au particles (Supporting Note 2 and Supporting Figure 4b). To confirm the successful encapsulation of Au particles within the polymer matrix, X-ray fluorescence (XRF) measurements were employed. Figure 2b,c shows the Au mass and the number of Au particles on the crystal violet-Au particles-treated polymers, respectively. The Au weight percentage (wt %) in the crystal violet-Au particles-treated polymer, as measured by XRF, was used to calculate the Au mass or particle number in the treated polymer. The polymer treated with 9.9 nm Au particles contained a significantly higher Au mass than other samples, while the polymer treated with 3.7 nm particles had a lower Au mass than other samples. When the Au mass was converted into the number of particles, the polymer treated with 1.2 nm Au particles had the highest particle number. In contrast, the sample treated with 8.6 nm Au particles had the lowest particle number.

**Figure 2:**
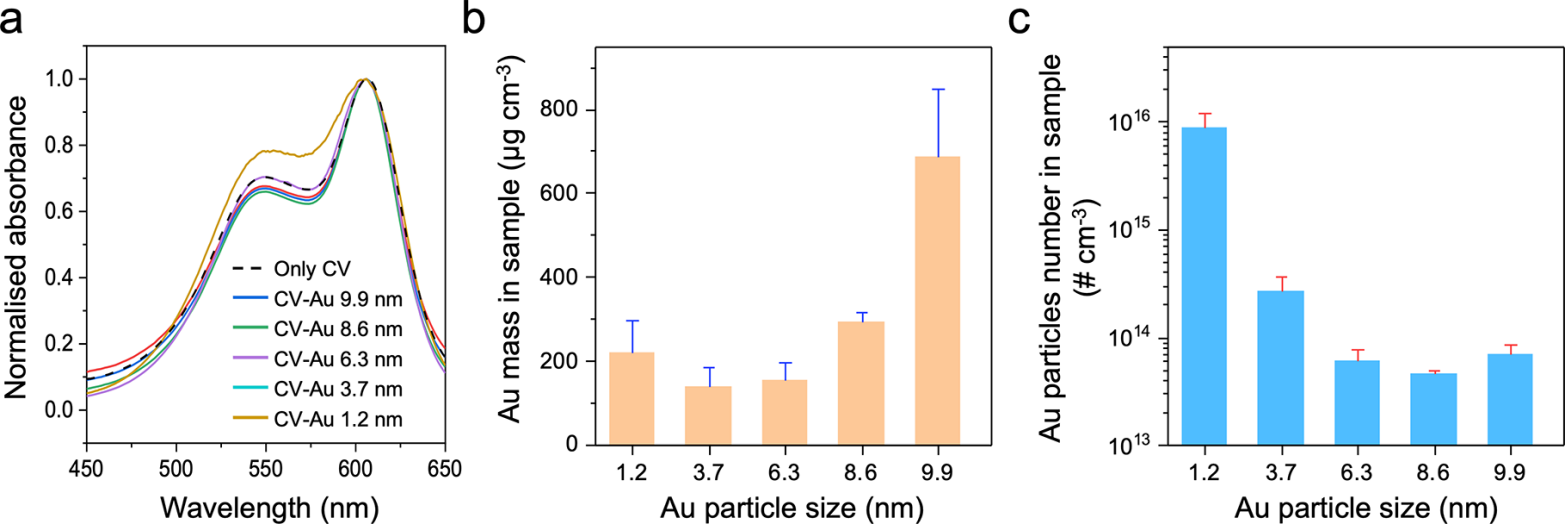
(a) UV–vis absorbance spectra of crystal violet (CV)-treated polymer surfaces with 1.2, 3.7, 6.3, 8.6, and 9.9 nm Au particles in the wavelength range of 450–650 nm. In this measurement, intact silicone polymer was used as a reference to account for background absorption from the polymer matrix. (b) Au mass and (c) the number of Au particles within the treated polymers, determined by using XRF.

Figure 3a shows a Jablonski diagram explaining the photoreaction mechanism of the organic photocatalyst. With the absorption of visible light, the crystal violet molecule goes from a ground state (S_0_) to an excited singlet state (S_1_) with a paired electron spin. Crystal violet in S_1_ returns to S_0_ or transforms to a triplet state (T_1_) with an unpaired electron spin. The molecule in T_1_ undergoes two different photochemical reactions known as redox reactions associated with the generation of O_2_•^–^, H_2_O_2_, ^•^OH, and energy transfer leading to the generation of ^1^O_2_.^50^ Photoluminescence (PL)/phosphorescence (PH) spectroscopies and time-resolved electron paramagnetic resonance (tr-EPR) spectroscopy were employed to investigate the size effect of Au particles on photoexcited charge carriers’ behavior in crystal violet. Based on the emission wavelength of the visible light source and the light absorption property of crystal violet, the excitation wavelength was selected between 500 and 600 nm (Figure 2a and Supporting Figure 5). Figure 3b shows the steady-state PL spectra of the treated polymer samples, measured in the wavelength range of 600–780 nm at an excitation of 530 nm. The addition of Au particles ≥ 8.6 nm did not alter the PL spectrum. However, adding Au particles ≤ 6.3 nm to the crystal violet-treated polymer reduced the PL peak intensity, with the most pronounced decrease observed for 1.2 nm Au particles.

**Figure 3:**
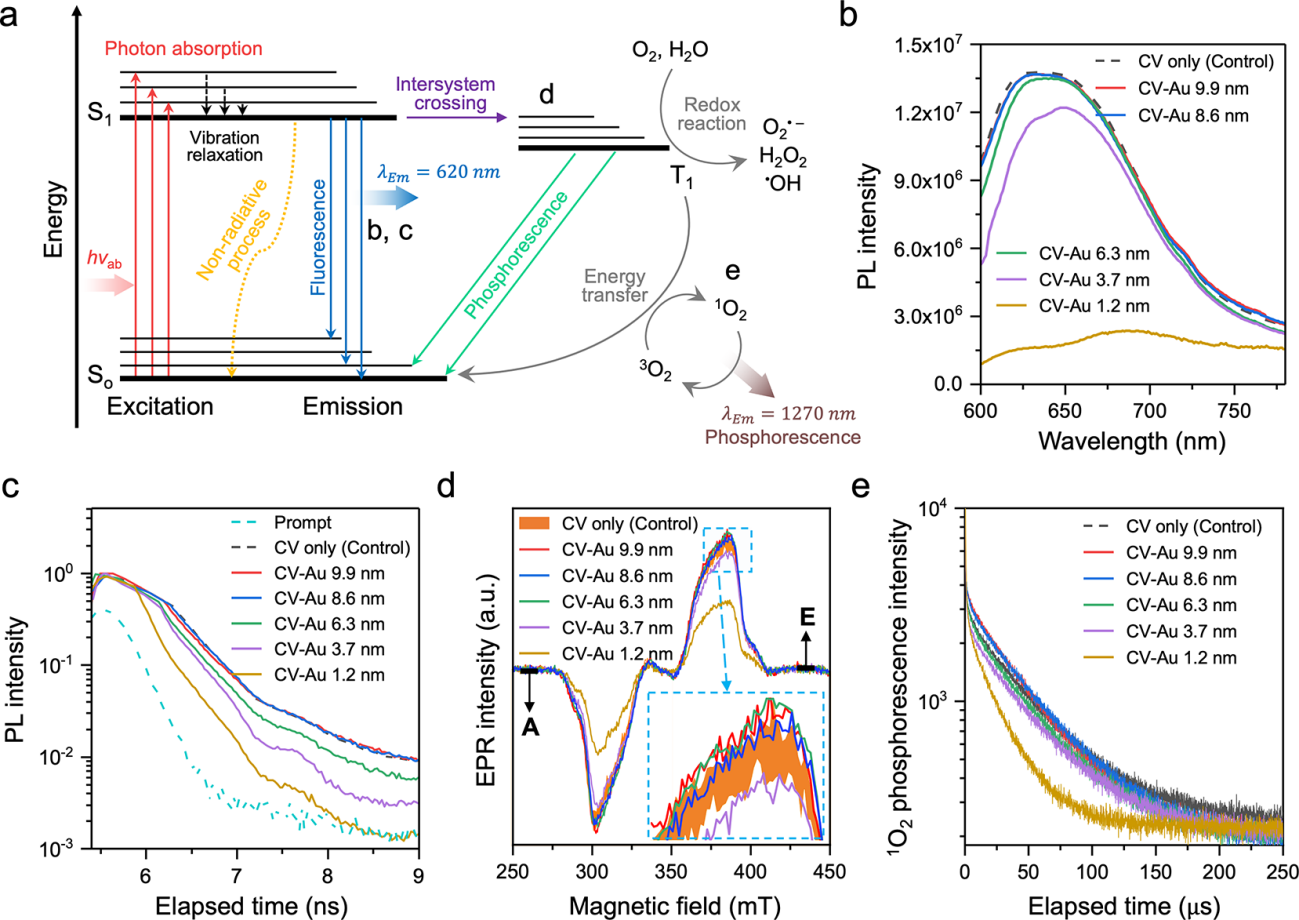
(a) Jablonski diagram showing the photoreaction mechanism of crystal violet (CV). (b) Steady-state photoluminescence (PL) spectra (λ_Ex_ = 530 nm), (c) time-resolved PL decay (λ_Ex_ = 502 nm, λ_Em_ = 620 nm) and (d) time-resolved electron paramagnetic resonance spectra of CV before and after addition of 1.2, 3.7, 6.3, 8.6, and 9.9 nm Au particles (λ_Ex_ = 600 nm). For the polymer with crystal violet only, measurements were performed on two different samples to evaluate the reproducibility of the experiment; the shaded area represents the range between the two spectra. A and E indicate absorptive and emissive features, respectively. (e) Time-resolved ^1^O_2_ phosphorescence decay of CV before and after the addition of the Au particles (λ_Ex_ = 532 nm, λ_Em_ = 1270 nm).

In addition, polymers with Au particles only (no crystal violet) did not produce PL spectra under identical conditions (Supporting Figure 6). Thus, they were not further tested in tr-EPR, PL, and ^1^O_2_ PH spectroscopies. The PL lifetime decay measurement of the crystal violet-treated polymer after Au particle addition (λ_Ex/Em_ = 502/620 nm) was conducted (Figure 3c). The results showed no effect on PL lifetime with Au particles ≥ 8.6 nm. In contrast, with Au particles ≤ 6.3 nm, the PL lifetime decay was enhanced as the particle size decreased, indicating more efficient charge carrier transfer from crystal violet to Au particles. As shown in Figure 3d, changes in the T_1_ population of crystal violet after the addition of Au particles to the photocatalyst-treated polymer were observed. The tr-EPR spectra are consistent with the presence of spin-polarized triplet states of organic aromatic molecules that are non-Boltzmann populated.^51^ The profiles of all spectra were similar, but their intensity differed. The T_1_ population of crystal violet was not changed with Au particles ≥ 6.3 nm. However, reductions in the T_1_ population were observed on the polymers with Au particles ≤ 3.7 nm, indicating charge carrier transfer at S_1_ from crystal violet to the particles. Time-resolved PH spectroscopy further determined the consequences of the T_1_ population reduction. As shown in Figure 3e, the ^1^O_2_ PH lifetime decay of the crystal violet-treated polymer after Au particle addition mirrored the PL lifetime decay and T_1_ population trend, indicating that T_1_ reduction diminishes energy transfer to form ^1^O_2_.

To understand the mechanism of charge carrier transfer, the energy levels of crystal violet and Au particles were calculated using DFT and eqs 1 and 2. The citrate ligands used for Au particles ≥ 3.7 nm stabilize the particles by providing a negatively charged and protective layer.^52^ As a surface ligand, it acts as an insulating barrier, and its shell consists of organic functional groups, including carboxylate and hydroxyl moieties, which are poor electronic conductors.^53,54^ As shown in Supporting Note 3, in the case of 1.2 nm Au particles with semiconductive features, the lowest unoccupied molecular orbital (LUMO) is localized on the gold core, whereas the highest occupied molecular orbital (HOMO) is localized on the cysteine ligands, indicating that the cysteine ligands act as barriers to electron conduction rather than enhancing charge carrier mobility. Thus, it is appropriate to apply the band alignment calculation, which takes into account the ligand effect rather than the ligand-induced electronic coupling. Band alignments based on electron affinity (EA), ionization potential (IP), and Fermi levels (*E*_F_) show the interface of crystal violet, and 3.7 or 6.3 nm Au particles have an energy band of a metal/semiconductor junction. For the 1.2 nm Au particle with a discontinuous band structure, the energy band corresponds to a straddling gap heterojunction (Supporting Figure 11).^55–57^ In addition, no evidence of band junction formation between crystal violet and Au particles ≥ 8.6 nm was observed, as indicated by the lack of variations in the PL, PH, and tr-EPR spectra, suggesting that charge carrier transfer is not facilitated in these larger systems. Figure 4a illustrates the charge transfer mechanism between crystal violet and 6.3, 3.7, or 1.2 nm Au particles based on their energy levels with respect to the absolute vacuum scale (AVS) computed by DFT. It is suggested that the charge carrier transfer at T_1_ in the combination of crystal violet and 6.3 nm Au particles is attributed to the upward bending of bands of crystal violet (Figure 4a and Supporting Figure 11a). This spatial band bending could suppress the charge transfer at S_1_, making T_1_ a more favorable transfer site_._ In contrast, for crystal violet–3.7 nm Au particles, the absence of the spatial barrier allows the carrier transfer to occur at both S_1_ and T_1_ (Figure 4a and Supporting Figure 11b). For crystal violet–1.2 nm Au particles, the potential energy level of T_1_ in the photocatalyst is insufficient to facilitate charge carrier transfer from T_1_ to the LUMO band of the Au particles (Figure 4a and Supporting Figure 11c). Consequently, charge transfer predominantly occurs at the S_1_. These align with the spectroscopic results presented in Figure 3. Upon photoinduced electron transfer, crystal violet forms a radical cation (CV^+^•) characterized by a highly reactive hole. It is expected that due to the insufficient potential level of its S_0_, the hole cannot be neutralized via water oxidation.^58,59^ Instead, CV^+^• undergoes molecular degradation through redox interactions with ROS, leading to dissipation of the charge, indicating that the hole does not contribute to ROS generation.^60,61^ The degradation has been consistently observed in previous studies involving crystal violet in conjunction with 2 nm Au particles or silica–alumina supports.^38,62^ In addition, although Au particles exhibit surface plasmon resonance (SPR) under visible light, SPR might not play a dominant role in interfacial charge carrier dynamics or ROS generation in this study. This could be attributed to three factors. First, the S_1_ level of crystal violet is higher than the E_F_ of Au particles (Figure 4a), and the *E*_F_ shifts downward with increasing size, indicating the increased gap of energy levels between them.^63^ Second, the excited state lifetime of crystal violet (up to several nanoseconds for S_1_ and microseconds for T_1_) is significantly longer than that of Au particles (1–5 ps).^64–66^ Third, efficient ROS generation via SPR on Au particles typically requires highly intense light sources such as X-rays or a pulsed visible light laser.^67^

**Figure 4:**
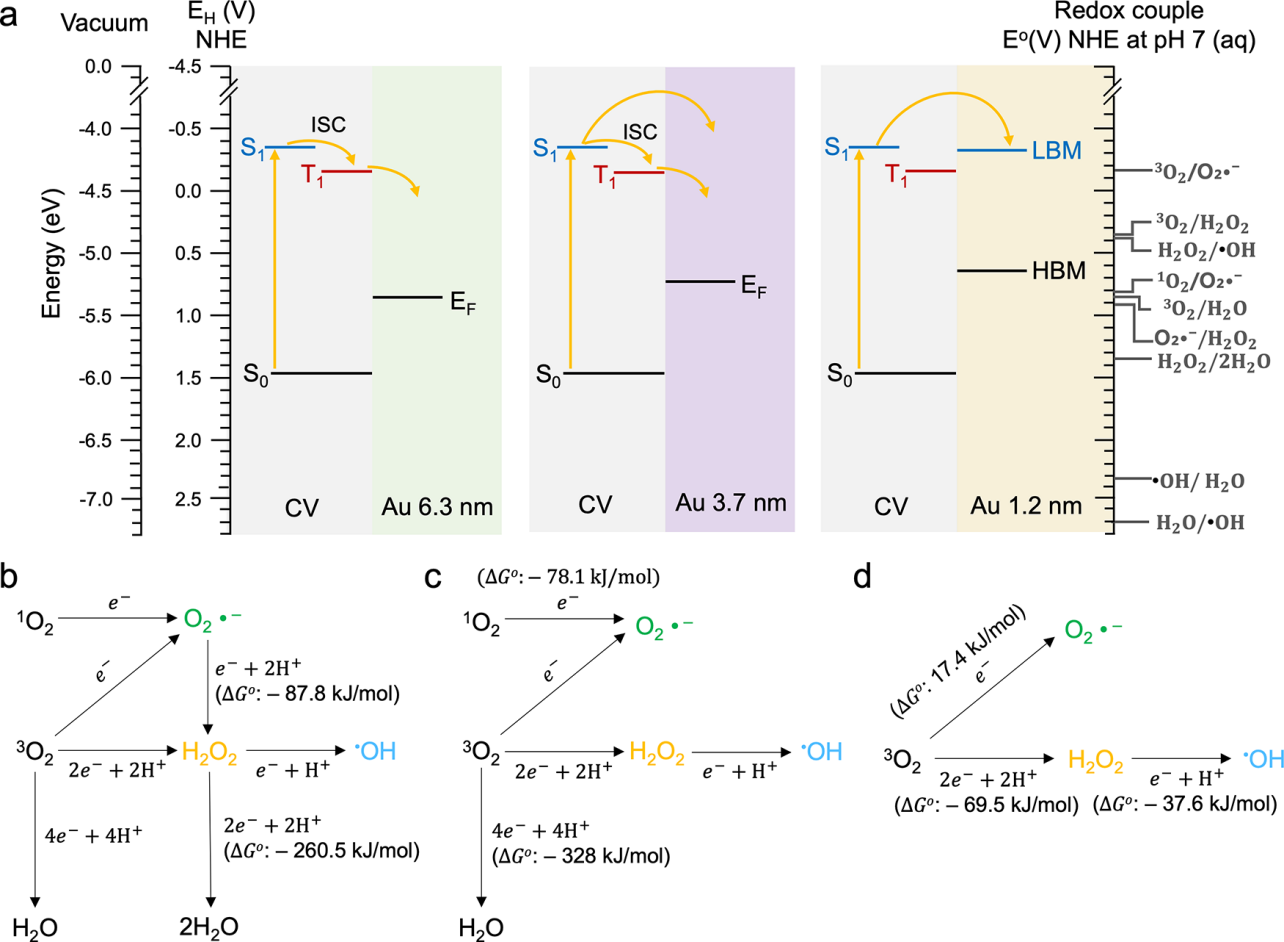
(a) Mechanism of charge carrier transfer between crystal violet (CV) and Au particles. Potential energy level diagram illustrating the ground state (S_0_), excited singlet state (S_1_), and excited triplet state (T_1_) of CV as the donor, alongside the Fermi levels (*E*_F_) of 6.3 and 3.7 nm Au particles, and LUMO band minimum (LBM) and HOMO band maximum (HBM) of 1.2 nm particles (acceptor) in aqueous condition, obtained by density functional theory (DFT) calculations and eqs 1 and 2. The energy scale is drawn with respect to the normal hydrogen electrode (NHE) and the absolute vacuum scale (AVS) as references. On the right side, redox couple potentials (E°) for ROS generation are presented with respect to NHE at pH 7 in aqueous condition.^58,59^ (b–d) Pathways of ROS generations in the combination of CV only, CV-6.3 nm Au particles, and CV-3.7 or 1.2 nm Au particles. Δ*G* ^*o*^ indicates Gibbs free energy changes.

Figure 4b–d shows the suggested reaction pathways of ROS generation on crystal violet only and the crystal violet-Au particles combination. Aligning the S_0_, S_1_, T_1_, E_F_, HOMO band maximum (HBM), LUMO band minimum (LBM), and E_H_ of the materials and redox couple potentials (E°) can identify whether ROS generation reactions are thermodynamically favorable (Figure 4a).^58,59,68^ It is suggested that the S_1_ and T_1_ levels of crystal violet can reduce molecular oxygen to generate O_2_•^–^, H_2_O_2_, ^•^OH, and H_2_O through e-, 2e-, and 4e- pathways (Figure 4b). However, its S_0_ level is insufficient to oxidize H_2_O to ^•^OH, which is a hole effect. The addition of Au particles ≤ 6.3 nm to crystal violet significantly improves ROS generation pathways. At the combination of crystal violet and 6.3 nm Au particles, H_2_O_2_ is exclusively produced via a 2e- pathway, and oxygen dissociation from H_2_O_2_ to H_2_O is incapable (Figure 4c). In the case of 1.3 or 3.7 nm Au particles, O_2_•^–^, H_2_O_2_, ^•^OH are reduced from ^3^O_2_ via e- and 2e- pathways, without reduction to H_2_O (Figure 4d).^58,59^ These changes could enhance the efficiency of ROS generation by minimizing the unnecessary side reactions or energy loss.

To determine whether the addition of Au particles to the photocatalyst enhanced the ROS generation through redox reactions, 2,3-bis­(2-methoxy-4-nitro-5-sulfophenyl)-5- [(phenylamino)­carbonyl]-2H-tetrazolium (XTT) sodium salt (100 μM) for O_2_•^–^, a quantitative peroxide assay kit for H_2_O_2_, and disodium terephthalate (110 μM) for ^•^OH was used.^68–70^ The samples were irradiated with a white light LED emitting visible light with an intensity range from 0.66 to 3.84 mW cm^–2^ (Supporting Figure 5). The optimal time for irradiation of the samples was selected to minimize the natural effect on ROS generation (Supporting Note 4). After 96 h exposure to visible light, the CV-treated polymer with 1.2 nm Au particles exhibited the highest O_2_•^–^ concentration (2.6 μM) of the tested samples, followed by the treated polymers with the 3.7 (1.1 μM) and 6.3 nm (0.7 μM) particles, respectively (Figure 5a). Similar trends were observed for H_2_O_2_ and ^•^OH (Figure 5b,c). After 6 h of exposure to visible light, the concentrations of H_2_O_2_ were 14, 8.6, and 6.0 μM on CV-treated polymers with 1.2, 3.7, and 6.3 nm Au particles, respectively. Adding 1.2 nm Au particles to the CV-treated polymer led to significantly higher ^•^OH generation than other samples. After 48 h of exposure to visible light, the concentration reached 0.523 μM, 33% higher than the total ^•^OH produced by other samples. In a dark room, ROS generation was not observed on any of the tested polymers (Supporting Figure 16–18). The decrease of O_2_•^–^, H_2_O_2_, and ^•^OH generation by the addition of Au particles ≥ 8.6 nm to the crystal violet-treated polymer can be attributed to the very low efficiency (<0.67%) of the interfacial charge transfer, thereby inhibiting the photocatalyst’s surface redox reactions.

**Figure 5:**
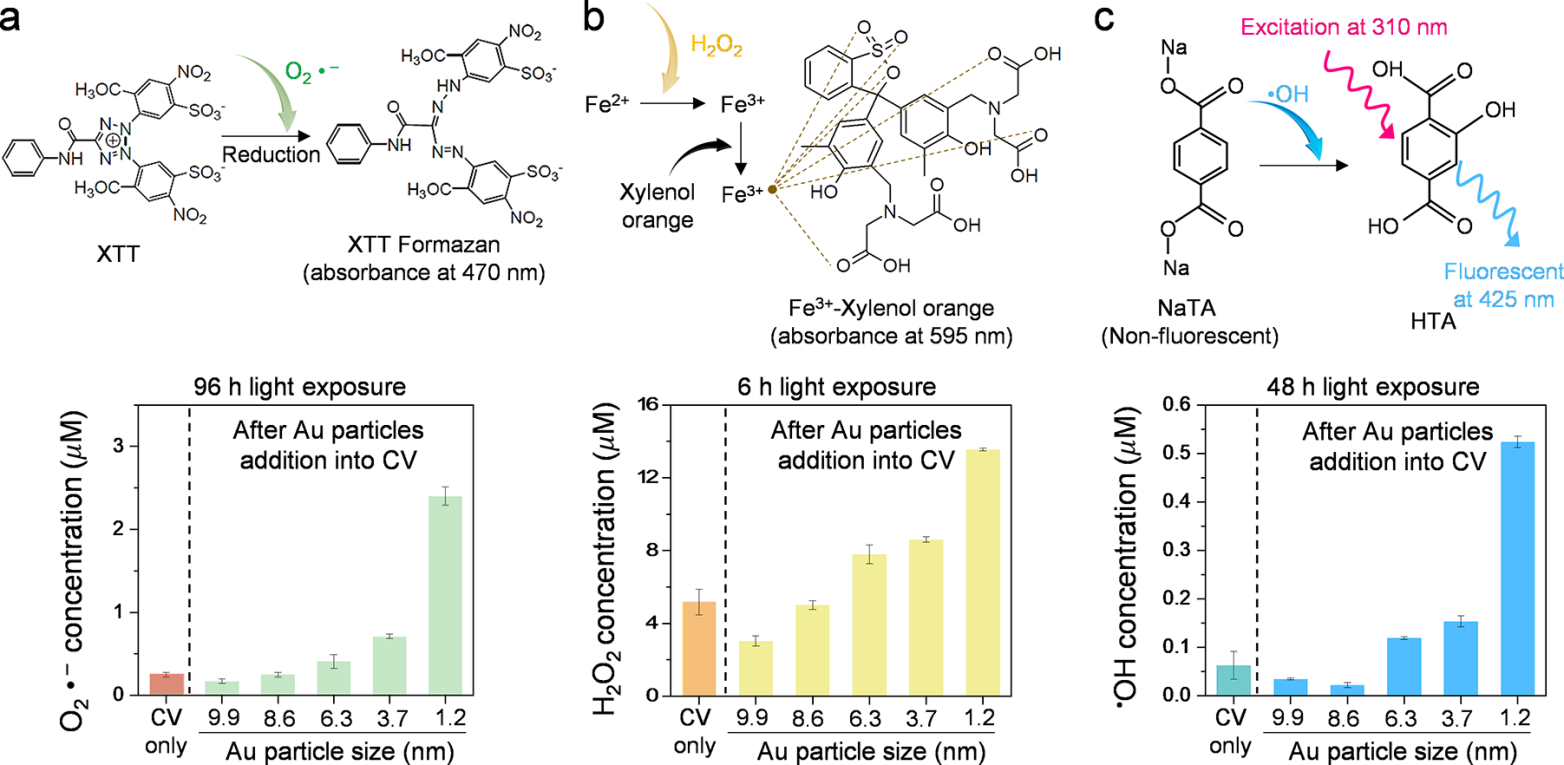
(a) Estimate concentration of O_2_•^–^ generated from crystal violet (CV) Au particles treated polymer surfaces after 96 h exposure to visible light (*n* = 3 independent samples). The inset shows the structural conversion of XTT sodium salt to XTT formazan upon reduction by superoxide radical (O_2_•^–^), resulting in increased absorbance at 470 nm. (b) Estimate concentration of H_2_O_2_ generated from CV–Au particles treated polymer surfaces after 6 h exposure to visible light (*n* = 3 independent samples). The inset shows the formation of the Fe^3+^-xylenol orange complex from Fe^2+^ in the presence of H_2_O_2_, causing an increase in absorbance at 595 nm. (c) Absolute concentration of ^•^OH generated from CV–Au particles treated polymer surfaces after 48 h exposure to visible light (*n* = 3 independent samples). The inset shows the formation of 2-hydroxyterephthalic acid (HTA) from disodium terephthalate acid (NaTA) via reaction ^•^OH, which results in increased fluorescence at 425 nm. Data are presented as mean ± SD.

The photodisinfection of crystal violet-Au particle-treated polymers in a hospital setting was tested against under low-intensity hospital lighting ranging from 0.037 to 0.054 mW cm^–2^ (Figure 6a). As shown in Figure 6b, disinfection tests were conducted in visible light and in a dark room. The polymer with crystal violet only was observed to have a disinfection activity with a 0.4 log reduction in the number of viable bacteria after 6 h incubation in the dark room, indicating that crystal violet retained its intrinsic disinfection property after the treatment.^49^ It was statistically confirmed that adding Au particles to the crystal violet-treated polymers did not significantly enhance disinfection activity in the dark (One-way ANOVA: *P*-value >0.1). Similarly, after 6 h exposure to visible light, no statistical difference in photodisinfection activities between polymers with crystal violet only and those with Au particles ≥ 8.6 nm was observed. (One-way ANOVA: *P*-value >0.1). However, the treated polymers with Au particles ≤ 6.3 nm showed a size-dependent enhancement in photodisinfection activity. The photodisinfection activities of the treated polymers with 3.7 and 6.3 nm Au particles were 0.8 and 1.2 log reductions in viable bacteria numbers, respectively, after 6 h exposure to visible light. The crystal violet-treated polymer with 1.2 nm Au particles represented the most potent photodisinfection activity with a 5.3 log reduction in the viable bacteria number. Considering the doping mass of 1.2 nm Au particles (Figure 2b), the disinfection efficiency was maximized with minimal materials usage. The visible light intensities in most healthcare facilities or other indoor settings remain at <0.15 mW cm^–2^ (Supporting Table 3). Therefore, lighting conditions must be carefully considered when designing photodisinfection surfaces for indoor applications. To validate the photodisinfection activity of the crystal violet-treated polymer with 1.2 nm Au particles, its efficiency was compared to the results of previous studies regarding the *Staphylococcus* spp. strain (Supporting Table 4)^24–26,33,37–39,62,71–80^ As shown in Figure 6c, many photodisinfection surfaces reported in previous studies can only be applied to limited areas of healthcare facilities, such as working tables in operation rooms, or are not realistic because the use of an intense light source is required. However, the crystal violet-1.2 nm Au particle-treated surface, which has potent disinfection activity at up to 6000 times lower visible light intensities or even the lowest light dose (Supporting Figure 19), can be effectively applied in most ambient light conditions found in hospital settings.

**Figure 6:**
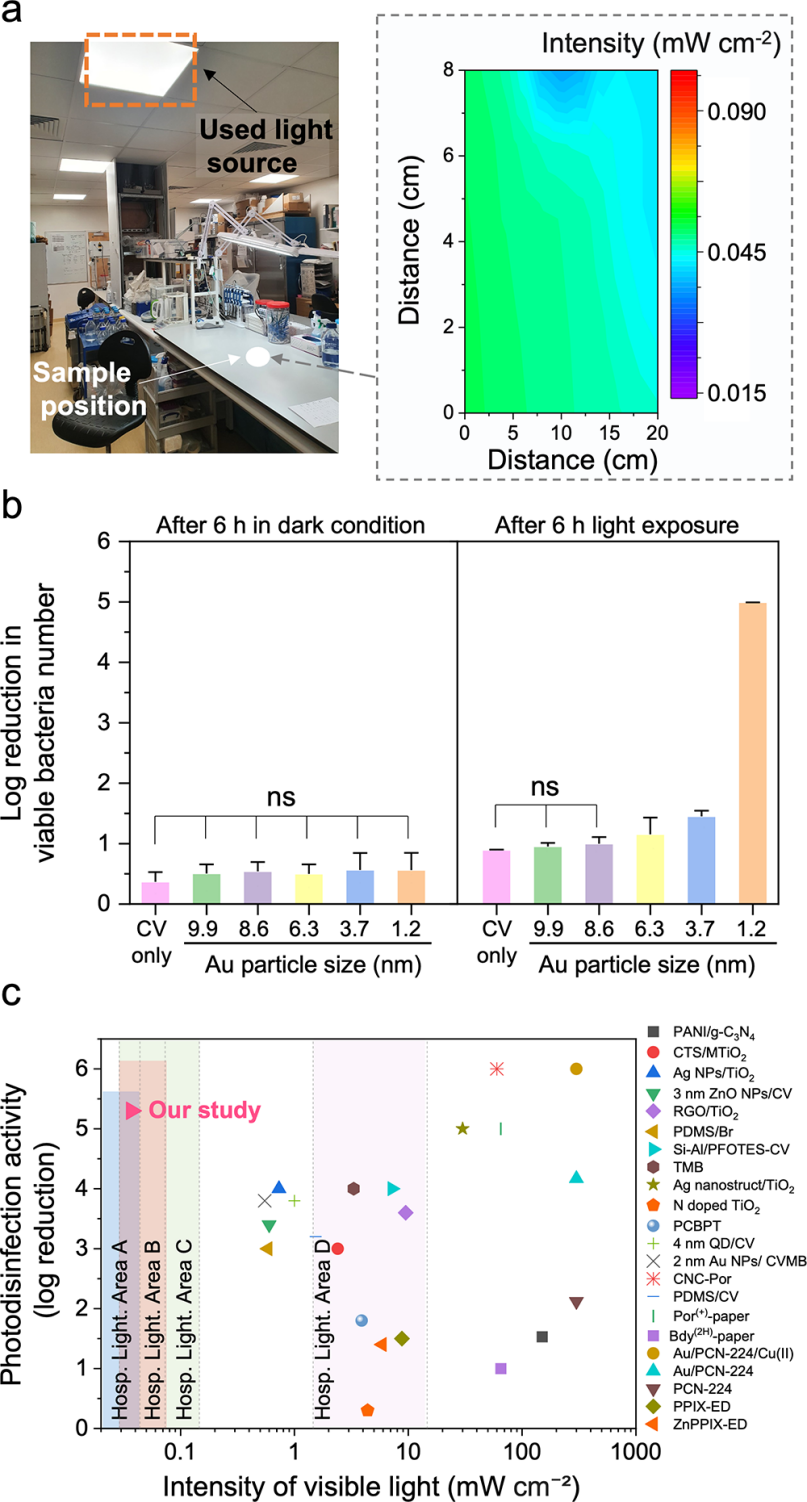
(a) Image of the real hospital lighting setting, where the antimicrobial tests were conducted. The graph in the gray box shows the intensity distribution of visible light over the area where the test samples were positioned. The visible light intensity ranged from 0.037 to 0.054 mW cm^–2^. (b) Disinfection activities of crystal violet (CV) only and CV–Au particles treated samples in visible light and in dark. “ns” means not statistically significant (*P*-value > 0.1, *n* = 6 independent samples). Data presented as mean ± SD (c) Comparison of the photodisinfection activity of various photobiocidal surfaces against *Staphylococcus* spp. strains regarding visible light intensities.^24–26,33,37–39,62,71–80^ The color shaded in the graph shows the recommended lighting intensity for different hospital areas. The blue shaded area (Hosp. Light. Area A) for wards, waiting rooms, and nurse stations. The orange shaded area (Hosp. Light. Area B) for ICU, investigation, and treatment rooms. The green shaded area (Hosp. Light. Area C) for exam room, nurse station and investigation/treatment room. The pink shaded area (Hosp. Light. Area D) for working tables in operation rooms. Supporting Tables 3 and 4 show detailed information about lighting guides for healthcare facilities and the photodisinfection results of the surfaces above, respectively.

This technique was further applied to a keyboard cover, one of the most frequently touched surfaces by healthcare workers, transforming it into a photodisinfection surface. As shown in Figure 7, the modified keyboard cover exhibited potent photodisinfection properties, achieving a > 5 log reduction in the number of viable under ambient visible light conditions in a hospital setting. Regarding the synthetic reproducibility of the technique, two key pieces of evidence support its consistency. First, the coefficient of variation for the UV–vis absorbance spectra of the crystal violet-treated polymer embedded with 1.2 nm Au particles was calculated to be 5.6%, indicating minimal variability in the synthesis. Second, as shown in Figure 6b, the photodisinfection activity consistently demonstrated a 5.3-log reduction in viable bacterial counts across all six samples, confirming the reproducibility of the disinfection performance. These findings demonstrate a high degree of reproducibility in the encapsulation process and the functional performance of the treated polymer. These findings suggest that this technique can be extended to various polymer-based hospital devices, such as catheters, screen covers, mobile phone and tablet covers, and endotracheal tubing, without compromising disinfection efficiencies observed when transitioning from laboratory conditions to real-world applications.^81^

**Figure 7:**
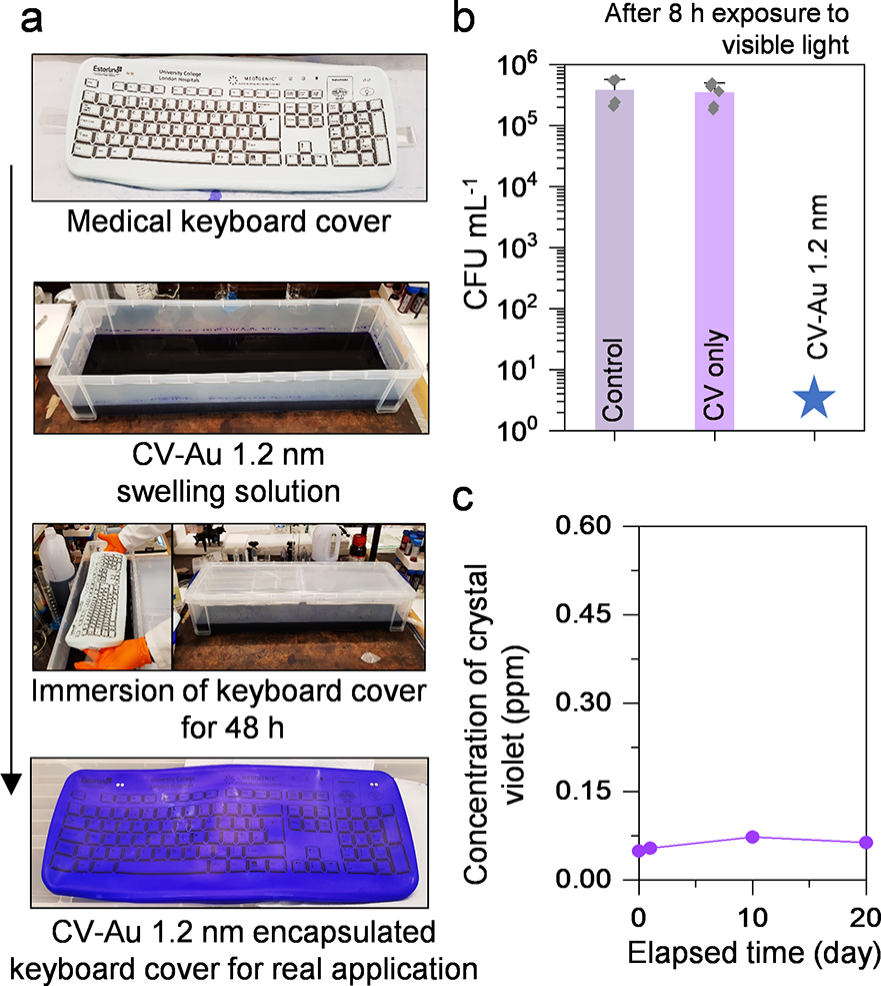
(a) Transformation from Medigenic keyboard cover to photodisinfection keyboard cover through an encapsulation process. (b) Photodisinfection activity of the keyboard cover after 8 h exposure to ambient visible light in a hospital setting. Blue star: no bacterial colony counts observed. (c) Leaching of crystal violet (CV) molecules from the treated keyboard cover over a 20-day exposure period. Small piece of the cover (1.5 cm × 1.5 cm) was immersed in 5 mL of PBS.

## Conclusions

To the best of our knowledge, for the first time, the present study investigated changes in photocatalytic reaction on crystal violet, an organic photocatalyst, by reducing the size of Au particles from nano to cluster region under visible light. The addition of Au particles ≥ 8.6 nm into the crystal violet-treated polymer did not improve photoexcited charge carrier transfer. In comparison, the use of Au particles ≤ 6.3 nm promoted charge carrier transfer from crystal violet to Au particles, which act as electron acceptors. Photospectroscopy analyses and DFT computations revealed that a change in the electronic band structure caused by the size reduction of the particle altered the charge carrier transfer pathway in crystal violet. Especially for crystal violet–1.2 nm Au particles, charge carrier transfer predominantly occurs at the S_1_ of crystal violet because the potential energy level of T_1_ in the photocatalyst is insufficient to facilitate the transfer. Au particles ≤ 6.3 nm on crystal violet significantly enhanced the generation of O_2_•^–^, H_2_O_2_, and ^•^OH by minimizing unnecessary side reactions or energy loss, while ^1^O_2_ generation decreased with reducing the size of the Au particle. The photodisinfection tests against *S. aureus* under a real hospital lighting setting showed that the disinfection activity was enhanced with Au particles ≤ 6.3 nm on the treated polymer. The most potent disinfection was observed on the crystal violet-Au 1.2 nm particle-treated polymer, which resulted in a 5.3 log reduction in viable bacteria after 6 h of exposure to visible light. This work provides valuable information for a fundamental understanding of the Au effect as a cocatalyst on photocatalysts and to develop a light-activated self-sterilizing surface that can be applied to various hospital surfaces to prevent the spread of pathogens responsible for HAIs, which remains a global challenge.

## Experimental Methods

### Synthesis of Au Particles Ranging from 3.7 to 9.9 nm in Size

Au particles ranging from 3.7 to 9.9 nm in size were synthesized using a seeded-growth method.^44^ The synthesis was performed in a three-neck round-bottom flask mounted on a hot plate (Stuart UC152D). A condenser was mounted on the flask, and a bath circulator pumped cooling water (10 °C) through the condenser (Pilot One thermoregulator, Huber, DE). To synthesize a seed Au particle, which is 3.7 nm in size, a mixture consisting of 50 mL of sodium citrate (Na_3_C_6_H_5_O_7_, 2.2 mM, Sigma-Aldrich), 33 μL of tannic acid (C_76_H_52_O_46_, 2.5 mM, Sigma-Aldrich) and 0.33 mL of potassium carbonate (K_2_CO_3_, 150 mM, Sigma-Aldrich) was heated at 70 °C under constant stirring. When the mixture reached the desired temperature, 0.33 mL of tetrachloroauric acid (HAuCl_4_, 25 mM, Sigma-Aldrich) was added to the reaction vessel, resulting in the solution color changing from colorless to black. The reaction was kept under constant stirring for 7 min, where the solution color turned dark red, indicating the formation of Au particle seeds. To grow the seeds to 6.3 nm, 1/3 (18.3 mL) of the seed solution was removed from the reaction vessel and replaced with an equal volume of sodium citrate solution (2.2 mM). The mixture was heated for 5 min to reach 70 °C, and then 0.165 mL of 25 mM HAuCl_4_ was added to the reaction vessel. The addition of HAuCl_4_ to the reaction vessel was repeated until the required 6.3, 8.6, and 9.9 nm Au particles were obtained. Prior to the seeded-growth process, we confirmed that the Au particle seeds were well dispersed without any agglomeration using HRTEM (Supporting Figure 1b).

### Synthesis of 1.2 nm Au Particles

1.2 nm Au particles, which are [Au_25_(Cys)_18_] clusters, were synthesized using a tube-in-tube reactor system, which was previously introduced by Gavriilidis’ research group.^45^ 45.5 mg of l-cysteine was added into 5 mL of distilled (DI) water, 9.9 mL of HAuCl_4_ solution (25.2 mM, Sigma-Aldrich), and 0.83 mL of sodium hydroxide solution (NaOH, 2 M, Sigma-Aldrich) were added to the cysteine solution, and then 9.27 mL of DI water was added into the mixture. With a gaseous CO supply, the prepared Au precursor solution was delivered to a tube-in-tube reactor at a flow rate of 0.33 mL/min via a Teflon AF-2400 tube using a milliGAT LF pump (VICI Valco). The inner temperature of the reactor was maintained at 80 °C. During the 3 min travel of the precursor through the reactor, 1.2 nm Au particles were synthesized and then collected at the reactor’s outlet.

### HRTEM and UV–Vis Absorbance Spectroscopy for Au Particles

To confirm the size and shape of synthesized Au particles ranging from 1.2 to 9.9 nm in size, HRTEM (Titan G2 Cube 60-300, FEI) was used. A droplet of the particle solutions was inoculated on the TEM grid and dried in the air. The images of the particles were taken at an accelerating voltage of 80 kV. To determine particle size, 20 images under each condition were analyzed using ImageJ software. However, the uniformity of particle distribution or density was not evaluated because during drying, the coffee right effect and capillary flow alter the original distribution state of the particles on the TEM grid.^82–84^ Thus, it is concluded that an accurate assessment of uniformity and density is not feasible under these conditions. UV–Vis absorption spectra of Au particle solutions were measured at wavelengths of 350–800 nm using a UV–vis-NIR spectrophotometer (UV-3600i Plus, Shimadzu).

### ESI–MS

To confirm that the 1.2 nm Au particles are [Au_25_(Cys)_18_] clusters, the dried particles were mixed with DI water with 0.01 μM cesium acetate. The particle solution was supplied into an ESI-MS instrument (Q-TOF 6510, Agilent Technologies) at a flow rate of 20 μL min^–1^ using a syringe pump (KD Scientific). The electrospray ionization was operated in negative mode, and heated nitrogen, the drying gas, was supplied to the spectrometer at a flow rate of 5 L min^–1^. The negatively charged ions were analyzed by the mass spectrometer.

### Production of Crystal Violet-Au Particle-Treated Polymer Surface

In a mixture of acetone (100 mL), Au particles (20 mL), DI water (80 mL), or acetone (100 mL) and DI water (100 mL), crystal violet (Sigma-Aldrich) was dissolved at a concentration of 800 ppm. A silicone polymer sheet (4.0 cm × 7.0 cm) was immersed in the mixture for 24 h. The polymer sheet was collected from the solution, washed with DI water twice to eliminate unbound materials, and then air-dried for 24 h in a dark room. In addition, polymer sheets with only Au particles were prepared using the sample process without crystal violet.

### UV–Vis Absorbance Spectroscopy for the Treated Polymer Surface

UV–Vis absorbance spectra of crystal violet only treated polymer and crystal violet treated with Au particles were measured in wavelengths of 450–700 nm using a UV–vis-NIR spectrophotometer (UV-3600i Plus, Shimadzu).

### XRF Spectroscopy

The weight percentage (wt %) of gold within the treated polymers was measured using an XRF spectrometer (15 W Epsilon 4, Malvern Panalytical Ltd., U.K.) to calculate the Au mass or the number of Au particles in the polymers. The treated polymer (1.5 cm × 1.5 cm) was placed into a pot with a colorless substrate film; they were placed into an XRF spectrometer, and then the elements were measured.

### Steady-State and Time-Resolved PL Spectroscopies

The PL spectra of the treated polymers (1.5 cm × 1.5 cm) were measured in wavelengths of 600–780 nm using a steady-state PL spectrometer (FluoroMax, Horiba Scientific) at the excitation wavelength of 530 nm. The PL lifetime decays of the treated polymers (1.5 cm × 1.5 cm) were measured using a Fluorolog spectrometer with a time-correlated single photon counting (TCSPC) apparatus (Horiba Scientific). Pulsed 502 nm excitation (100 MHz repetition rate, <140 ps pulse width) was generated by a laser diode (DeltaDiode, Horiba Scientific), and the fluorescence was detected at wavelengths of 620 nm for time scales up to 100 ns (∼26.7 ps resolution). The decay curves were normalized to maximum PL intensity to compare changes in PL lifetimes before and after the addition of Au particles to the crystal violet-treated polymer.

### tr-EPR Spectroscopy

X-band (9.6 GHz/0.34 T) tr-EPR spectra were recorded on a Bruker Elexsys E580 spectrometer equipped with an ER 4118X-MD5 dielectric resonator (Bruker) and a SpecJet-III signal digitizer (Bruker). For all of the experiments, a microwave power of 3 mW was used. The measurements were performed at a temperature of 50 K; this was set using a closed-circuit helium cryostat (Cryogenic Ltd.) controlled by a Lake Shore 350 ITC (Lake Shore Cryotronics, Inc.). Pulsed sample photoirradiation was performed through the optical window of the cryostat, made from Spectrosil B, by using an Aurora II Integra laser system (Litron Lasers Ltd.). This consists of a Q-switched Nd:YAG laser (∼6 ns pulse width and 21 Hz repetition rate) coupled with an optical parametric oscillator (OPO). The OPO was tuned for an output wavelength of 600 nm (∼5 mJ per pulse). Each sample (0.2 × 1 cm) was loaded into a clear fused quartz (CFQ) tube with an inner diameter of 3 mm and a wall thickness of 1 mm (Wilmad 707-SQ-100M). Before the measurement, the sample surface was aligned to be normal to the laser beam; for this purpose, a quartz tube with an inner diameter of 1 mm and a wall thickness of 1 mm (Wilmad 712-SQ-100M) was placed against the back side of the sample to prevent spontaneous reorientation during the measurements. The 2D tr-EPR data sets were processed using MATLAB scripts written in-house; the spectra displayed in Figure 3d were extracted at the time position yielding the maximum signal.

### Time-Resolved ^1^O_2_ PH Spectroscopy

To measure ^1^O_2_ PH, a near-infrared sensitive thermoelectrically cooled photomultiplier was used. The treated polymer (1.5 cm × 1.5 cm) was placed onto a slide glass and irradiated by a Nd:YAG laser operating at 532 nm. A PC-mounted multiscaler board with preamplifier (MSA-300, Becker-Hickl) was used as the photon counting system to measure ^1^O_2_ phosphorescence lifetime decay at a wavelength of ∼1270 nm. Using FluoFit software (PicoQuant GmbH), the ^1^O_2_ lifetime decay curves were normalized so that the phosphorescence peak was set to 10^4^ counts.

### Fermi Levels of Au Particles >1.2 nm

The Fermi level shift (Δ*E*_F_) for Au particles was calculated using^63^

$$\Delta E_{F}\approx \left(\frac{2}{3}\right)E_{F}\left(\frac{3\times r}{R}\right)$$ 1

where *E*_F_ is the Fermi level of gold bulk at normal temperature,^85,86^ *r* is the radius of gold particles, and *R* is the Thomas–Fermi screening length of gold.^87^ The Fermi level of each size of the particles was calculated using

$$E_{\mathrm{FVAC}\;\mathrm{level}}^{\mathrm{Au}\;\mathrm{particle}}=E_{F\mathrm{VAC}\;\mathrm{level}}^{\mathrm{Au}\;\mathrm{bulk}}+\Delta E_{F}$$ 2

where *E*_F__VAC level_^Au particle^ indicates the Fermi level of Au particles with respect to the vacuum level, and *E*_F__VAC level_^Au bulk^ indicates the Fermi level of Au bulk with respect to the vacuum level.

### Computational Details

We employed the FHI-aims code for the density functional theory calculations performed in this study with the intermediate basis set, allowing the relativistic effect at the Zora level. Structures of the crystal violet molecule, whose overall charge was taken to be +1, considering the experiment was performed in an aqueous environment, and the 1.2 nm Au particle, modeled as a thiol-protected [Au_25_(Cys)_18_]. Atomic structures of the crystal violet molecule and the Au particle were optimized using the PBE (GGA) functional with a maximum residual force per atom below 0.5 × 10^–3^ eV Å^–1^. Afterward, their electronic structures were further optimized at the PBE0 level of theory to obtain IP and EA, including with and without the effect of solvent using the MPB method, simulating water at 300 K. To investigate the electronic excited states of a crystal violet molecule, we employed the delta self-consistent field (ΔSCF) method as implemented in the FHI-aims code.^88–90^

### Band Alignment of Crystal Violet and Au Particles

The band alignments between crystal violet and Au particles were calculated based on the *E*_F_, EA, and IP calculated by the density functional theory or eqs 1 and 2 above. For crystal violet and Au particles > 1.2 nm, the alignment of metal and semiconductor under equilibrium was applied.^51,91^ The bending degree of the crystal violet energy band (*V*_BB_) and Schottky barrier (ϕ_SB_) at the interface of the particle and photocatalyst were calculated using^56,91^

$$V_{\mathrm{BB}}=|\phi_{\mathrm{Au}}-\phi_{\mathrm{cv}}|$$ 3

where ϕ_Au_ and ϕ_cv_ indicate the Fermi levels of Au particles and crystal violet, respectively, and

$$\phi_{\mathrm{SB}}=\phi_{\mathrm{Au}}-\chi_{\mathrm{CV}}$$ 4

where χ_CV_ indicates the EA of crystal violet.

In the case of crystal violet and 1.2 nm Au particle, the band alignment of two semiconductors under equilibrium was used because the particle, Au_25_ cluster, has a discontinuous band structure.^35,55^ The bending degrees of energy bands of two semiconductive materials were calculated using eq 3, and offsets of EA (Δ*E*_EA_) and IP (Δ*E*_IP_) for the two materials were calculated using^55^

$$\Delta E_{\mathrm{EA}}=E_{\mathrm{EA}}^{\mathrm{CV}}-E_{\mathrm{EA}}^{\mathrm{Au}}$$ 5

and

$$\Delta E_{\mathrm{IP}}=E_{\mathrm{IP}}^{\mathrm{CV}}-E_{\mathrm{IP}}^{\mathrm{Au}}$$ 6

where *E*_EA_^CV^ and *E*_EA_^Au^ indicate the electron affinities of crystal violet and 1.2 nm Au particle, respectively, and *E*_EA_^CV^ and *E*_EA_^Au^ indicate the ionization potential of crystal violet and 1.2 nm Au particle, respectively.

### Superoxide Radical (O_2_•^–^) Measurement

XTT sodium salt (Sigma-Aldrich) was used at a concentration of 100 μM in DI water. The treated polymer was cut into small pieces with a diameter of 7 mm and placed at the bottom of wells with a diameter of 7 mm in 96-well plates. 250 μL of the XTT solution was added to each well, and the plate was covered with a lid. The plates were exposed to a white light LED lamp for 96 h, while another set of plates was kept in a dark room for 96 h. After that, the UV–vis absorbance spectra of the solutions collected from the plate were measured in wavelengths of 300–700 nm. For each UV–vis measurement, 750 μL of the XTT solution was collected from three samples. The O_2_•^–^ concentration was calculated using the Beer–Lambert eq 7 below.

$$C=\frac{A}{\varepsilon \times l}$$ 7

where *C* is the concentration of XTT formazan (M), resulting from an interaction of XTT sodium salt and O_2_•^–^, *A* is the absorbance at 470 nm, ε is the molar extinction coefficient (2.16 × 10^4^ M^–1^ cm^–1^ of XTT formazan at ∼470 nm), and *l* is the optical path length (cm).^68,92^ In addition, we did confirm that the absorbance value fell within the linear dynamic range of the assay, but we are not sure whether our experimental conditions precisely matched those under which the reported ε was originally determined. Thus, the quantification for O_2_•^–^ provides approximate rather than absolute concentrations.

### Hydrogen Peroxide (H_2_O_2_) Measurement

Pierce quantitative peroxide assay kit (Thermo Scientific), consisting of reagent A, which contains 25 mM ammonium ferrous (II) sulfate and 2.5 M sulfuric acid (H_2_SO_4_), and reagent B, which contains 100 mM sorbitol and 125 μM Xylenol orange in water, was used. Reagents A and B were mixed at a volume ratio of 1:100. The treated polymer was cut into small pieces with a diameter of 7 mm and placed at the bottom of a well with a diameter of 7 mm in 96-well plates. 250 μL of the peroxide assay solution was added to each well, and the plate was covered with a lid. The plates were exposed to a white light LED lamp for 48 h, while another set of plates was kept in a dark room for 48 h. After that, the UV–vis absorbance spectra of the solutions collected from the plate were measured in wavelengths of 450–850 nm. For each UV–vis measurement, 750 μL of the assay solution was collected from three samples. The H_2_O_2_ concentration was calculated using Beer–Lambert eq 7. In this case, *C* is the concentration of Fe^3+^-xylenol orange complex (M), which resulted from an interaction of the assay solution and H_2_O_2_, *A* is an absorbance at 595 nm, and ε is the molar extinction coefficient (1.5 × 10^4^ M^–1^ cm^–1^ of the complex at 595 nm, *l* is the optical path length (cm).^69^ For a reason similar to that for the O_2_•^–^ measurement, this also provides an approximate concentration.

### Hydroxyl Radical (^•^OH) Measurement

Disodium terephthalate acid (NaTA, Sigma-Aldrich) was used at a concentration of 110 μM in DI water. The treated polymer was cut into small pieces with a diameter of 7 mm and placed at the bottom of wells with a diameter of 7 mm in a 96-well plate. 250 μL of NaTA solution was added to each well, and the plate was covered with a lid. The plates were exposed to a white light LED lamp for 96 h, while another set of plates was kept in a dark room for 96 h. After that, the Photoluminescence spectra of the solutions collected from the plate were measured at wavelengths of 380–500 nm. For each measurement, 750 μL of the NaTA solution was collected from the three samples. NaTA interaction with ^•^OH forms 2-hydroxyterephthalic acid (HTA), which emits main fluorescence at 425 nm upon excitation at 310 nm.^70^ To calculate the absolute concentration of ^•^OH, 97% HTA (Sigma-Aldrich) was used, and a calibration curve was established by measuring fluorescence intensities at 425 nm, corresponding to the known concentrations of HTA (Supporting Figure 15).

### Gibbs Free Energy Change

Gibbs free energy changes were calculated for the ROS redox reaction using^58^

8 $$\Delta G^{o}=-nF\Delta E^{o}$$

where *n* is the number of electrons gained in redox couple reactions, *F* is the Faraday constant (96,485 C mol^–1^), and Δ*E* ^*o*^ is the electrode potential of ROS redox couple at pH 7 and 25 °C.

### Photobactericidal Test

The bactericidal activities of treated polymers were estimated against (NCTC 10788) in visible light and in a dark room. One 10 μL loopful of the bacterial plate-culture grown on Columbia blood agar (Oxoid, UK) was inoculated into nutrient broth and aerobically cultured at 37 °C with 200 rpm shaking for 18 h. The bacteria were harvested through centrifugation (20 °C, 1500 × *g* for 10 min), washed using 10 mL of sterile phosphate buffer saline (PBS), and then centrifuged to resuspend the bacteria in 10 mL of PBS. The bacterial suspension was diluted 10000-fold to obtain ∼10^5^ CFU mL^–1^. 25 μL of the bacterial suspension was inoculated onto the treated polymer surfaces, and then the polymers were placed in colorless Petri dishes. After that, the polymers were exposed to visible light, which was located on the ceiling of the hospital laboratory, while an identical set of polymers were placed in a dark room. The polymers were placed in 450 μL of PBS and vortexed for 1 min to resuspend the bacteria from the polymers into the PBS. After a serial dilution, 100 μL of the bacterial suspension was plated onto Columbia blood agar and cultured at 37 °C for 24 h. The bacterial colonies grown on the agar were counted.

### Statistical Analysis

A one-way analysis of the covariance test and Pearson correlation coefficient on experimental data were analyzed using the statistical functions of Microsoft Excel.

### Light Intensity Measurement

The intensity distribution of visible light was measured using a Lux meter (DL7040, Di-Log, U.K.). The recorded lux data was converted to values in mW cm^–2^ using^93^

$$w=\frac{i}{6830}$$ 9

where *w* represents the light intensity in mW cm^–2^ and *i* indicates the light intensity in lux.

## Supplementary Material







## Notes

TEM image of Au particles; ESI-MS analysis on 1.2 nm Au particles; UV–vis absorbance and steady-state PL spectra of polymer with Au particles only; light intensity and emission spectrum of white LED lamp; DFT computation of crystal violet and 1.2 nm Au particle; band alignment of crystal violet and Au particles; optimization of chemical trapping method for ROS; recommended guidelines of lighting; and comparison of visible light active surfaces introduced in previous studies (PDF)
Crystal violet optimized geometry file (XYZ)
1.2 nm Au particle optimized geometry file (XYZ)
