# Supplementary material for: Optimizing the Au Particle Doping Size for Enhanced Photocatalytic Disinfection under Low-Intensity Visible Light
Source: ACS Nano. 2025 Jul 24;19(30):27740–53. doi: 10.1021/acsnano.5c07650 (PMC12333410; doi:10.1021/acsnano.5c07650)
Supplement: Supplementary file 1 [file nn5c07650_si_001.pdf]

Supporting information

Optimizing Au Particle Doping Size for Enhanced Photocatalytic Disinfection Under Low-Intensity Visible Light

Gi Byoung Hwang[1,8], Ki Joon Heo[2,8], Woongkyu Jee[1], Luca Panariello[3], Jacopo Piovesan[3], Mabel Cornwell[3], Alberto Collauto[4], Andreas Kafizas[4], Shanom Ali[5], Caroline Knapp[1], Alexander J. MacRobert[6], Asterios Gavriilidis[3], Ivan P. Parkin[1], Scott M. Woodley[1], and Jae Hee Jung[7]*

[1]Department of Chemistry, University College London, 20 Gordon Street, London, WC1H 0AJ, United Kingdom

[2]School of Mechanical Engineering, Chonnam National University, 77 Yongbong-ro, Gwangju, 61186, Republic of Korea

[3]Department of Chemical Engineering, University College London, Torrington Place, London, WC1E 7JE, United Kingdom

[4]Department of Chemistry, Imperial College London, Molecular Science Research Hub, White City Campus, 82 Wood Lane, London W12 OBZ, United Kingdom

[5]Environmental Research Laboratory, University College London Hospitals NHS Foundation Trust, 235 Euston Road, London, United Kingdom, NW1 2BU

[6]UCL Division of Surgery and Interventional Science, Royal Free Campus, Rowland Hill Street, London NW3 2PF, UK

[7]Department of Mechanical Engineering, Sejong University, 209 Neungdong-ro, Seoul, 05006, Republic of Korea

[8]These authors contributed equally

[*] Corresponding author: e-mail: hwanggi2@gmail.com

Table of contents

1. Supporting Figure 1-- Page 1
2. Supporting Note 1 (Supporting Figure 2 & Table 1) --------------------- Page 2 − 3
3. Supporting Figure 3-- Page 3
4. Supporting Note 2 (Supporting Figure 4) ----------------------------------- Page 4
5. Supporting Figure 5 – 6 -- Page 5
6. Supporting Note 3 (Supporting Figure 7 – 10 & Table 2) --------------- Page 6 – 9
7. Supporting Figure 11--- Page 9
8. Supporting Note 4 (Supporting Figure 12 – 15) --------------------------- Page 10 – 12
9. Supporting Figure 16 – 18-- Page 13 – 15
10. Supporting Table 3 --- Page 16
11. Supporting Table 4 --- Page 17
12. Supporting Figure 19 -- Page 18
13. References--- Page 19 – 21

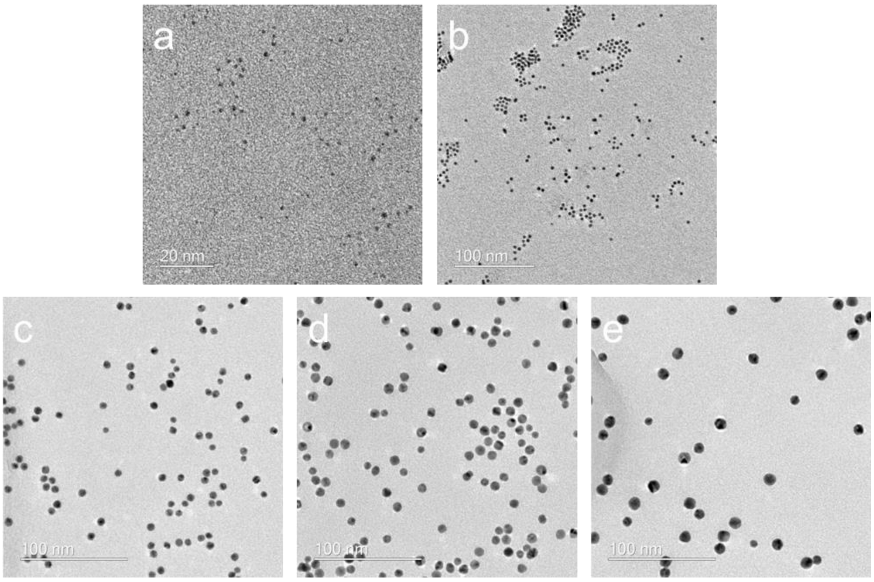

Supporting Figure 1. HRTEM images of (a) Au 1.2 nm, captured at a magnification of 100,000×; (b) Au 3.7 nm; (c) Au 6.3 nm; (d) Au 8.6 nm; and (e) Au 9.9 nm, captured at a magnification of 40,000×. In the case of 1.2 nm Au particles, the image was captured at higher magnification compared to other Au particles because it is difficult to recognize the small particles at a magnification of 40,000×.

Supporting Note 1

Supporting Figure 2 shows the zoomed-in ESI-MS of the isotopic patterns of peaks #1-7 in Figure 1c. #1 in Supporting Figure 2 shows the isotopic pattern of the peak spacing between ^{12}C and ^{13}C, which is 0.33, indicating that $[Au_{25}(Cys)_{18}]$ carried three negative charges resulting in the generation of $[Au_{25}(Cys)_{18}\text{-}3H]^{3-}$ ions under ESI conditions, which gave the molecular mass (MW) of $[Au_{25}(Cys)_{18}\text{-}3H]^{3-}$ at 7083.20 Da. A series of six similar less intense peaks after *m/z* 2361,43, #1, shown in Figure 1c, correspond to H^+ dissociation and Na^+ coordination to $[Au_{25}(Cys)_{18}]$ under negative mode ESI conditions (Supporting Figure 2). The ionized species (#2 to #7) were (#2) $[Au_{25}(Cys)_{18}\text{-}4H + Na]^{3-}$ (MW 7107.37 Da), (#3) $[Au_{25}(Cys)_{18}\text{-}5H + 2Na]^{3-}$ (MW 7129.35 Da), (#4) $[Au_{25}(Cys)_{18}\text{-}6H + 3Na]^{3-}$ (MW 7151.33 Da), (#5) $[Au_{25}(Cys)_{18}\text{-}7H + 4Na]^{3-}$ (MW 7173.31 Da), (#6) $[Au_{25}(Cys)_{18}\text{-}8H + 5Na]^{3-}$ (MW 7195.30 Da) and (#7) $[Au_{25}(Cys)_{18}\text{-}9H + 6Na]^{3-}$ (MW 7217.28 Da) (Supporting Table 1). All peaks corresponded to $[Au_{25}(Cys)_{18}]$ dissociated with H+ or coordinated with Na+, which is a common contaminant ion in ESI-MS. Supporting Table 1 summarizes the assigned *m/z* peaks and their empirical formula and gas-phase ions. The theoretical characteristic peak positions of $[Au_{25}(Cys)_{18}]$ were simulated based on empirical formulations of gas-phase ions formed under ESI-MS using Thermo Xcalibur software. The theoretical characteristic peak positions predicted by the software agreed well with the experiment's characteristic peak positions, identifying $[Au_{25}(Cys)_{18}]$.

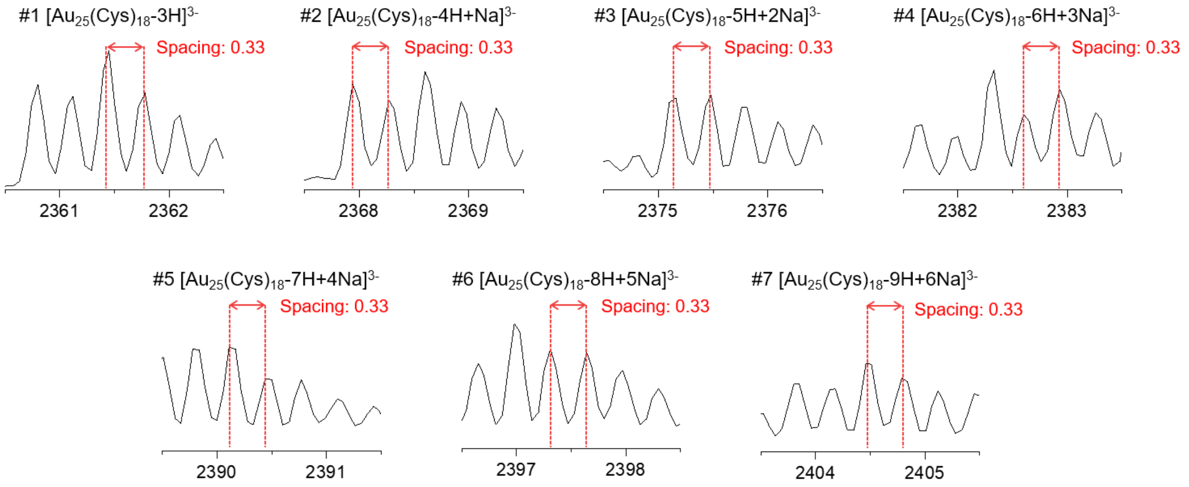

Supporting Figure 2. Enlarged experimental isotope patterns of peaks #1-#7 in Figure 1c.

Supporting Table 1 ǀ Theoretical and experimental characteristic peak positions of $[Au_{25}(Cys)_{18}]$ under ESI-MS.				
Peak number	Characteristic peak position (*m/z*)		Empirical formulations of gas-phase ions formed under ESI	Exact mass (Da)
	Theoretical	Experimental		
#1	2361.43	2361.45	$[Au_{25}(Cys)_{18}\text{-}3H]^{3-}$	7083.2
#2	2368.08	2367.94	$[Au_{25}(Cys)_{18}\text{-}4H+Na]^{3-}$	7107.37
#3	2375.44	2375.48	$[Au_{25}(Cys)_{18}\text{-}5H+2Na]^{3-}$	7129.35
#4	2382.74	2382.60	$[Au_{25}(Cys)_{18}\text{-}6H+3Na]^{3-}$	7151.33
#5	2390.08	2390.11	$[Au_{25}(Cys)_{18}\text{-}7H+4Na]^{3-}$	7173.31
#6	2397.42	2397.31	$[Au_{25}(Cys)_{18}\text{-}8H+5Na]^{3-}$	7195.30
#7	2404.76	2404.79	$[Au_{25}(Cys)_{18}\text{-}9H+6Na]^{3-}$	7217.28

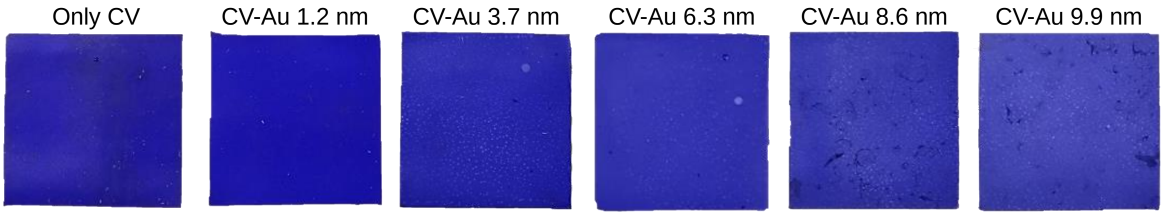

Supporting Figure 3. Images of the crystal violet (CV)-Au particles treated polymer surfaces.

Supporting Note 2

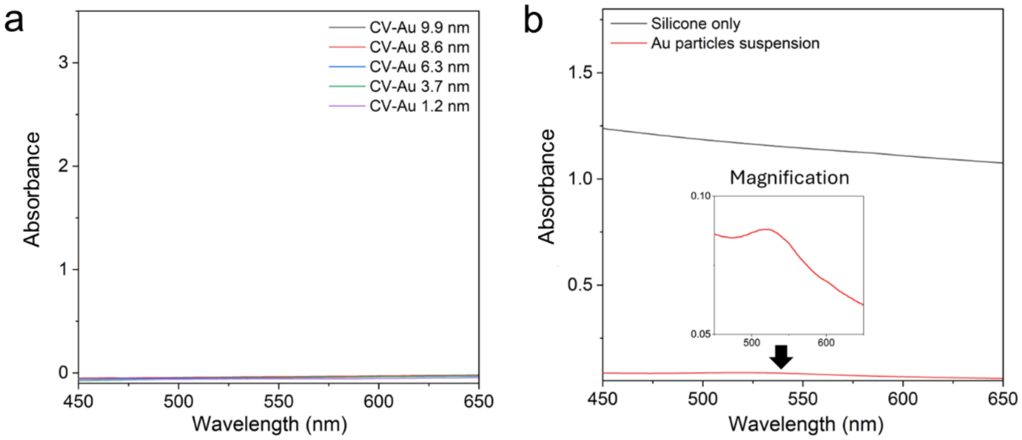

Supporting Figure 4. (a) UV-Vis absorbance spectra of polymers with Au particles only in wavelengths of 450 to 650 nm. In this measurement, intact silicone polymer was used as a reference to account for background absorption from the polymer matrix. (b) UV-Vis absorption spectra of silicone polymer alone and Au particles suspended in DI water at a concentration estimated from XRF analysis to match the highest Au content in the polymer.

An intact silicone polymer was used as a reference in the UV-Vis measurements for the treated polymer samples shown in Figure 2a and Supporting Figure 4a to account for background absorption from the polymer matrix. Supporting Figure 4b shows that the silicone exhibits an absorbance of ~1 in the 450–650 nm wavelength range. To enable meaningful comparison, the absorbance peak of the Au particle suspension was adjusted *via* dilution to match the maximum Au mass content (for 9.9 Au particles, Figure 2b) found in the polymer, as determined by X-ray fluorescence (XRF) analysis. Using the Beer-Lambert law, the expected absorbance was estimated. The adjusted absorbance peak was 0.08 at 510 nm, which is below the absorbance level of the silicone matrix at that wavelength. Previous studies have estimated Au particle uptake into polymers by measuring changes in the absorbance of the swelling solution before and after encapsulation, typically observing an absorbance change of approximately 0.04. Therefore, the adjusted absorbance peak value of 0.08 is consistent with expectations and is appropriate for this comparison [1]. These results indicate that the strong background absorption of the silicone polymer masks the characteristic absorption peak of Au particles. However, XRF analysis confirms that Au particles were successfully encapsulated within the polymer matrix (Figure 2b and c).

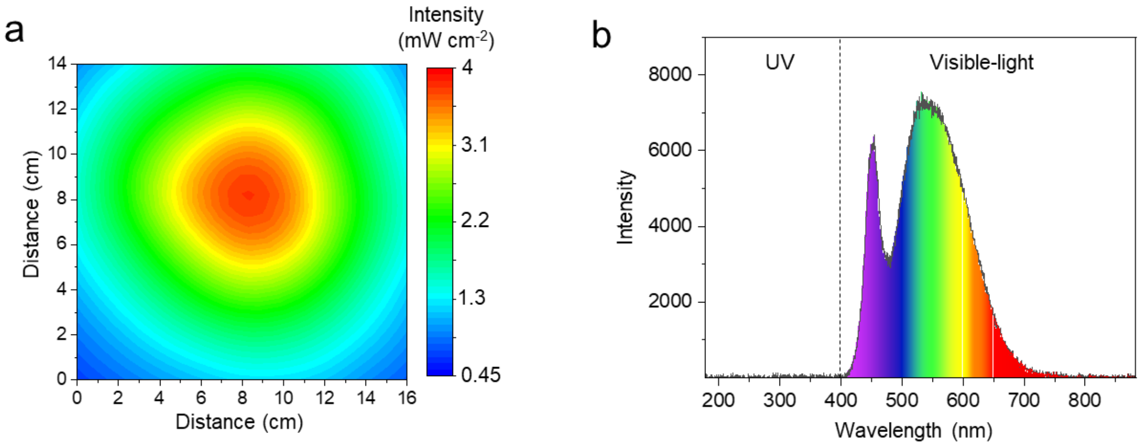

Supporting Figure 5. (a) Light intensity distribution of White LED lamp used in chemical trap assays, which measure the type and quantification of reactive oxygen species. (b) Emission spectrum of white LED lamp.

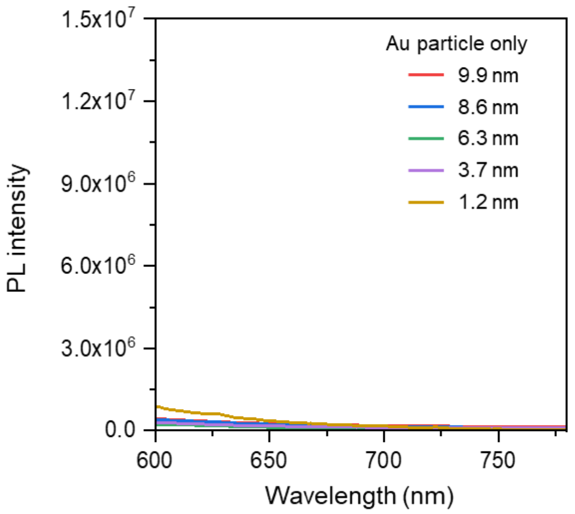

Supporting Figure 6. Steady-state photoluminescence spectra of polymer surfaces with Au particles only.

Supporting Note 3

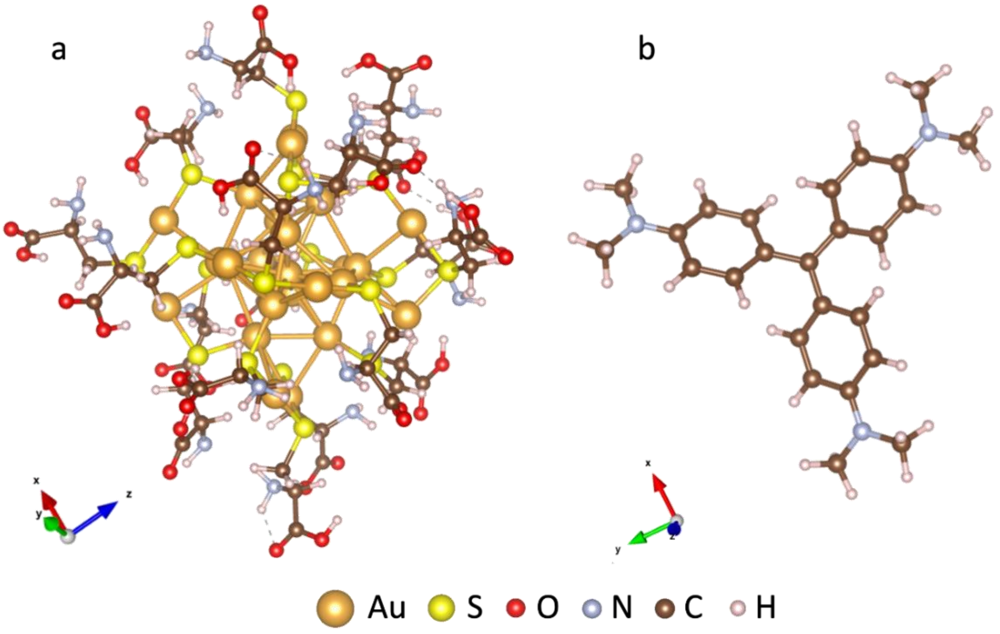

Supporting Figure 7. Geometry optimization performed at the GGA level of theory as implemented in the FHI-aims code. (a) 1.2 nm Au particle which thiol-protected Au_{25} cluster ($[Au_{25}(Cys)_{18}]$). (b) Crystal violet molecule. Coordinates of optimized structures are provided as Supporting files (xyz files).

We modelled a 1.2 nm Au particle which is thiol protected $[Au_{25}(Cys)_{18}]$. This particle is known for its well-established core-shell structure, where the core part consists of an Au_{13} icosahedral unit, surrounded by a shell of cysteine ligands and residual Au atoms [2-8]. It is important to note that the modelled cluster exhibits multiple local energy minima due to variations in the spatial orientation of the ligands. Therefore, we selected 16 lowest energy configurations or isomers of $[Au_{25}(Cys)_{18}]$ for detailed analysis. Supporting Figure 7 shows the tentative global minimum structure of $[Au_{25}(Cys)_{18}]$ obtained in this study along with the structure of the crystal violet molecule. Supporting Table 2 shows the computed electronic properties of the crystal violet molecule. Notably, the ionization potential (IP) and the electron affinity (EA) values show shifts in energy levels when solvent effects are included. This trend is consistent with the findings of our study and aligns with previous density functional theory (DFT) research. Moreover, the calculated EA in an aqueous environment is in close agreement with the experimental EA value of –3.13 eV [7]. We also computed the energy levels of excited singlet (S_1) and excited triplet (T_1) states for the crystal violet molecule, both in gas and aqueous phases. Supporting Table 2 provides the calculated energy levels of S_1 and T_1 where those values are referenced to the IP or the singlet ground state (S_0) of the crystal violet molecule. The calculated energy differences between the S_0 and S_1 or T_1 are 1.80 eV and 1.61 eV, respectively, which closely match the optical gap energy (~1.9 eV) reported in previous studies

[9]. Supporting Figure 8 presents the electronic energy states of the crystal violet molecule and 16 isomers of the modelled 1.2 nm Au particle. Although the values of EA, IP and Fermi-level (E_F) vary across the different Au particle isomers, it is noteworthy that these isomers can access their energetically neighboring local minima at room temperature [10, 11]. In this respect, the potential upper and the lower limits for the lowest unoccupied and highest occupied molecular orbital bands (LUMO and HOMO bands) of the model Au particle are –4.17 eV (LUMO band minimum, LBM) and –5.14 eV (HOMO band maximum, HBM), respectively. In particular, the energy level of LBM is bracketed between the energy levels of S_1 and T_1 of the crystal violet molecule, suggesting that the 1.2 nm Au particle function as electron acceptors.

The electronic ground-state charge distributions of the crystal violet molecule and the 1.2 nm Au particle were obtained using the same PBE0 level of theory, and the electron densities of selected states near the HOMO and LUMO levels were selected. Supporting figures 9 and 10 show total charge densities of HOMO and LUMO electronic states of crystal violet and 1.2 nm Au particle. The HOMO of crystal violet extends over phenyl rings and the central carbon connecting the three rings, suggesting high electron density over the aromatic system. LUMO is also spread across the triphenylmethane backbone, and the charge density is slightly shifted to a nitrogen atom on dimethylamino groups. In case of 1.2 nm Au particle, HOMO predominantly localized on cysteine ligand, with slightly extension to Au atoms whiles the LUMO is widely spread across Au atoms only, indicating that photoexcited charge carrier within crystal violet is transferred to Au atoms of the particle.

Supporting Table 2 | Calculated electronic properties of a crystal violet molecule in gas (g) and aqueous (aq) phases.

	Energy with respect to vacuum (eV)									
	IP		EA		S_1		T_1		E_F	
	(g)	(aq)	(g)	(aq)	(g)	(aq)	(g)	(aq)	(g)	(aq)
This work	−9.80	−5.94	−3.90	−2.92	−7.85	−4.14	−8.13	−4.33	−8.13	−5.34
DFT[a]	−9.08	−5.43	−4.27	−3.29	−6.61	-	−7.22	-	-	-

where IP and EA are ionization potential and electron affinity; S_1 and T_1 show singlet and triplet excited states, respectively; and E_F is the Fermi-level, see [a]Ref [12] .

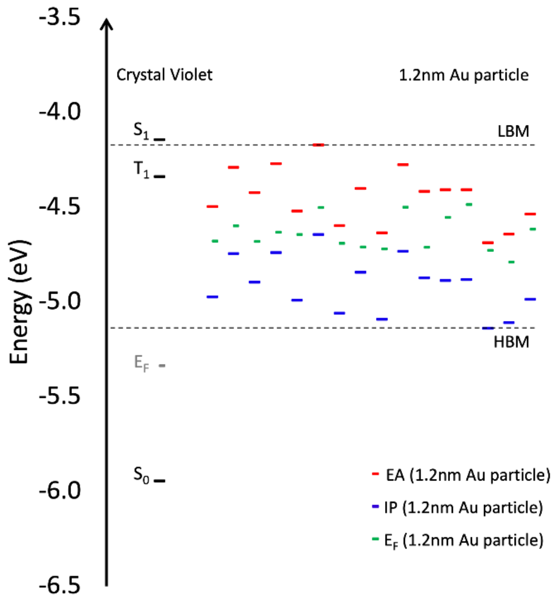

Supporting Figure 8. Calculated energy states of the crystal violet molecule and 1.2 Au particles in the aqueous phase**.** The ground singlet state (S_0), the excited singlet state (S_1), the excited triplet state (T_1) and the Fermi-level (E_F) of crystal violet**.** The electron affinity (EA), ionization potential (IP) and E_F for 16 isomers of the modelled 1.2nm Au particle. The stability of Au particle decreases from left to right: i.e., the values of EA, IP and E_F for the tentative global minimum/maximum clusters are shown at the far left-/right-hand sides. Horizontal dashed lines mark the LUMO band minimum (LBM) and the HOMO band maximum (HBM) of 1.2 nm particles.

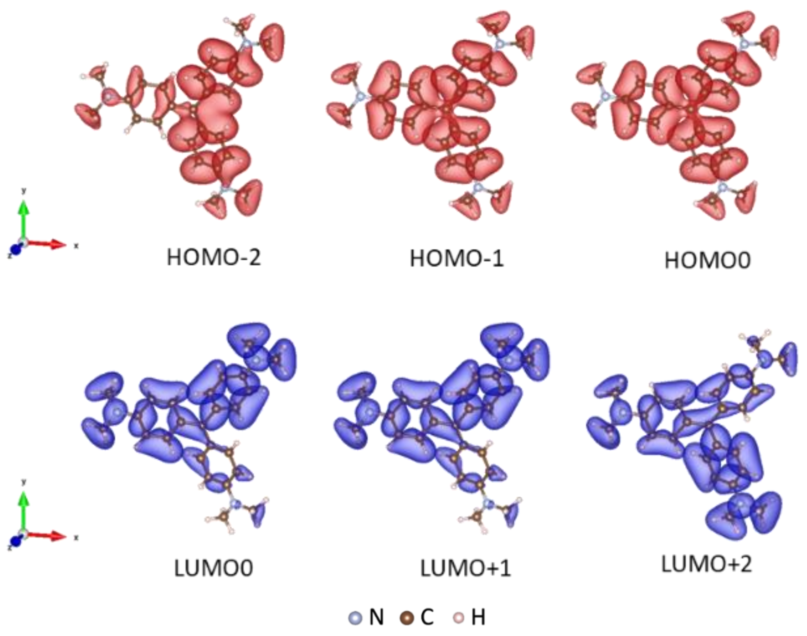

Supporting Figure 9. Total charge density of the electronic states of crystal violet molecule

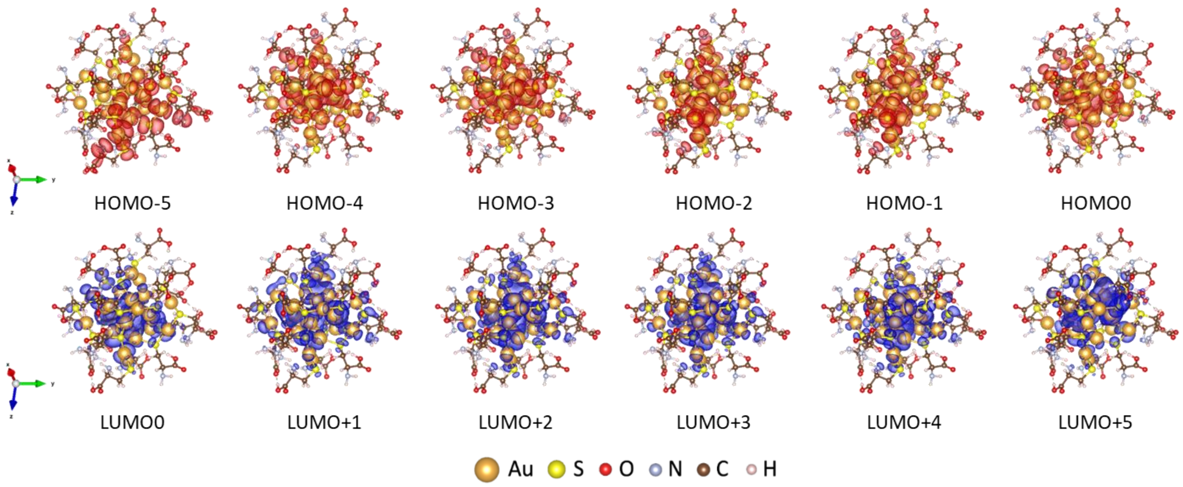

Supporting Figure 10. Total charge density of the electronic states of 1.2 nm Au particle which is [$Au_{25}(Cys)_{18}$].

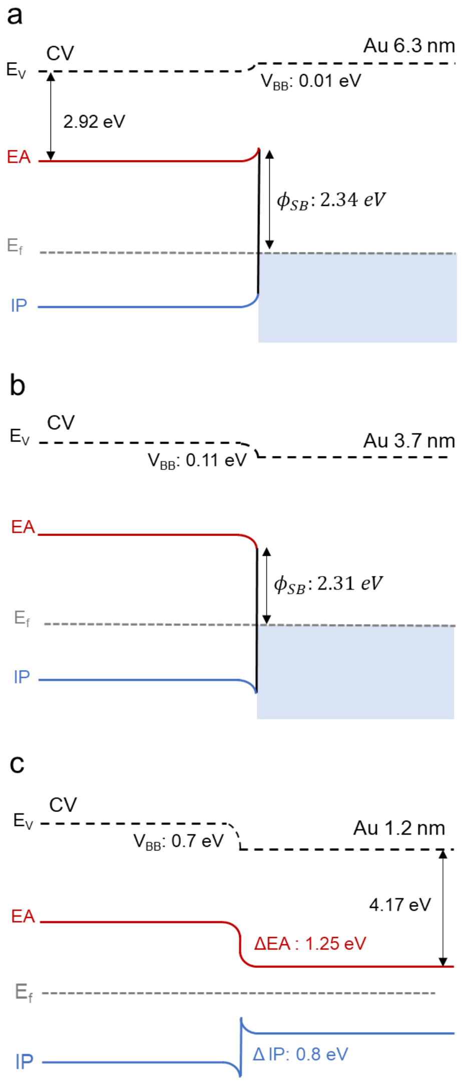

Supporting Figure 11. Band alignments of crystal violet (CV) and (a) Au 6.3, (b) 3.7 and (c) 1.2 nm particles based on Fermi level, electron affinity (EA) and ionization potential (IP) obtained by DFT computation. To calculate band alignments of CV and particles ≤ 6.3 nm, EA, IP, and E_F values obtained by DFT computations or equations (1) and (2) was used.

Supporting Note 4

To measure the concentration of $O_2{\bullet}^-$ generated by the samples in visible light, an XTT sodium salt solution (100 μM) was used. As shown in Supporting Figure 12, the UV-Vis absorbance spectra of XTT sodium salt solutions changed with increasing exposure time to visible light, and they were different depending on the samples. The absorbance values at 470 nm for each sample were subtracted from that of the control and then calculated using equation (7).

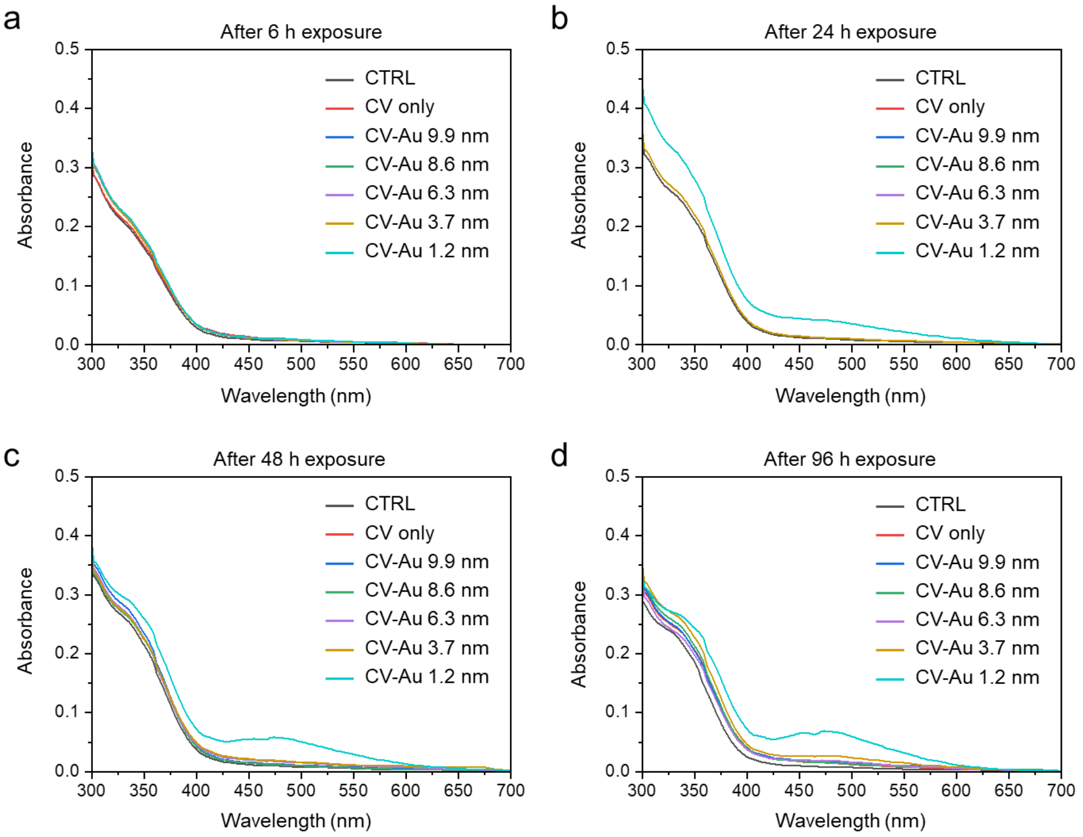

Supporting Figure 12. Change of UV-Vis absorbance spectra by superoxide radical ($O_2{\bullet}^-$) generated by control (XTT sodium salt solution), crystal violet (CV) only and CV-Au particles treated polymer surfaces after (a) 6, (b) 24, (c) 48, (d) 96 h exposures to visible light. XTT sodium salt solution, an $O_2{\bullet}^-$ chemical trap, was used.

To measure the concentration of H_2O_2 generated by the samples in visible light, a Pierce™ quantitative peroxide assay was used. As shown in Supporting Figure 13a, after 6 h exposure to visible light, the light absorption of the assay solution increased in wavelengths of 450 to 850 nm, and they were different depending on the samples, while the absorbance change for the control was not observed. After 24 or 48 h exposure to visible light, the absorbance change was not significant compared to that of the 6 h experiment. However, the absorbance of the control significantly increased after 24 h, suggesting that the intense light exposure for a prolonged time could cause xylenol orange to undergo peroxide-mediated oxidation (Supporting Figs. 13b and c). Thus, the 6 h condition was selected to determine the

concentration gap of H_2O_2 generated by different sizes of Au particles on the photocatalyst. The absorbance values at 595 nm for each sample were subtracted from that of the control and then calculated using equation (7).

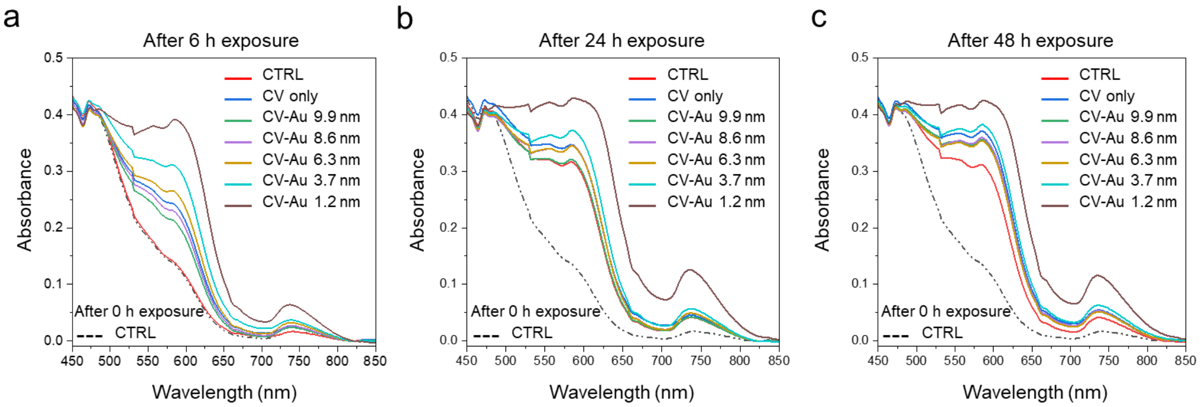

Supporting Figure 13. Change of UV-Vis absorbance spectra by hydrogen peroxide (H_2O_2) generated by control (Pierce™ quantitative peroxide assay solution), crystal violet (CV) only and CV-Au particles treated polymer surfaces after (a) 6, (b) 24, (c) 48 h exposures to visible light. Pierce™ quantitative peroxide assay, an H_2O_2 chemical trap agent, was used, and the spectra changes were measured in wavelengths of 450 to 850 nm.

To measure the concentration of •OH generated by the samples in visible light, a HTA (110 μM) solution was used. As shown in Supporting Figure 14, Photoluminescence (PL) was detected on crystal violet (CV)-treated polymer with 1.2 nm Au particles in the wavelength range of 380 to 500 nm after 24 h exposure to visible light, and the PL was detected on all tested samples after 48 h exposure. The PL increase on all samples after 96 h exposure to visible light was minor compared to the 48-h experimental result, while the PL increase in the control was significant. Thus, the 48-h condition was selected to determine the gap of •OH concentration. To determine •OH concentration, the PL values at 425 nm for each sample were subtracted from that of the control and then calculated using the calibration curve shown in Supporting Figure 15.

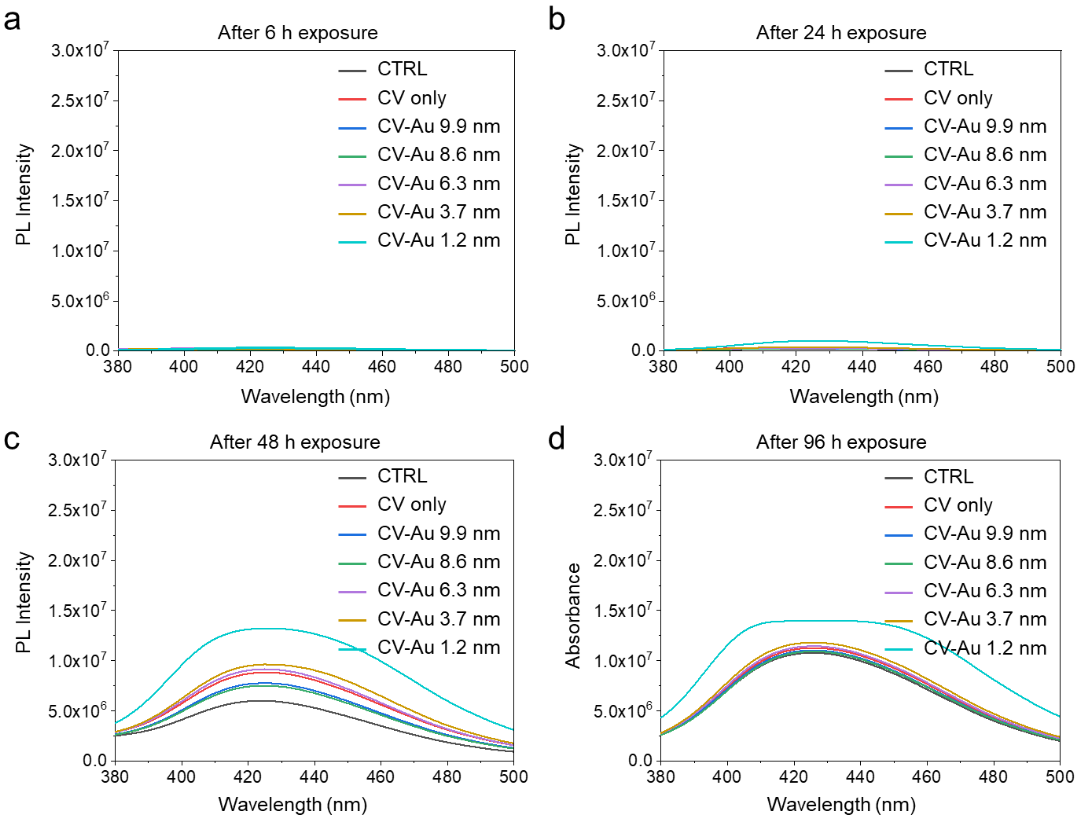

Supporting Figure 14. Change of photoluminescence (PL) spectra by hydroxyl radicals (•OH) generated by control (HTA solution), crystal violet (CV) only and CV-Au particles treated polymer surfaces after (a) 6, (b) 24, (c) 48, (d) 96 h exposures to visible light. A 2-hydroxyterephthalic acid (HTA) solution, an •OH chemical trap agent, was used, and the changes of the PL spectra were measured in wavelengths of 380 to 500 nm.

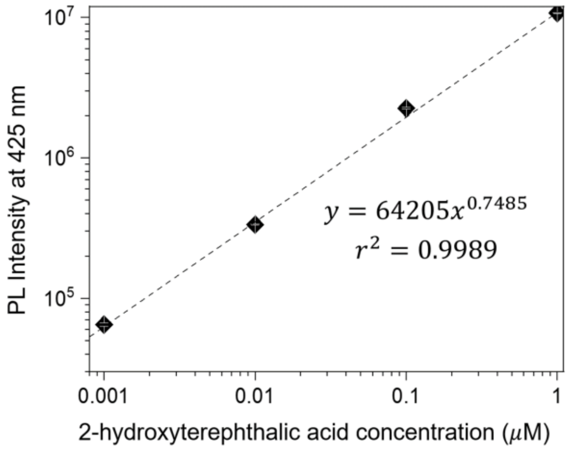

Supporting Figure 15. Correlation between 2-hydroxyterephthalic acid (HTA) concentration and PL intensity at 425 nm.

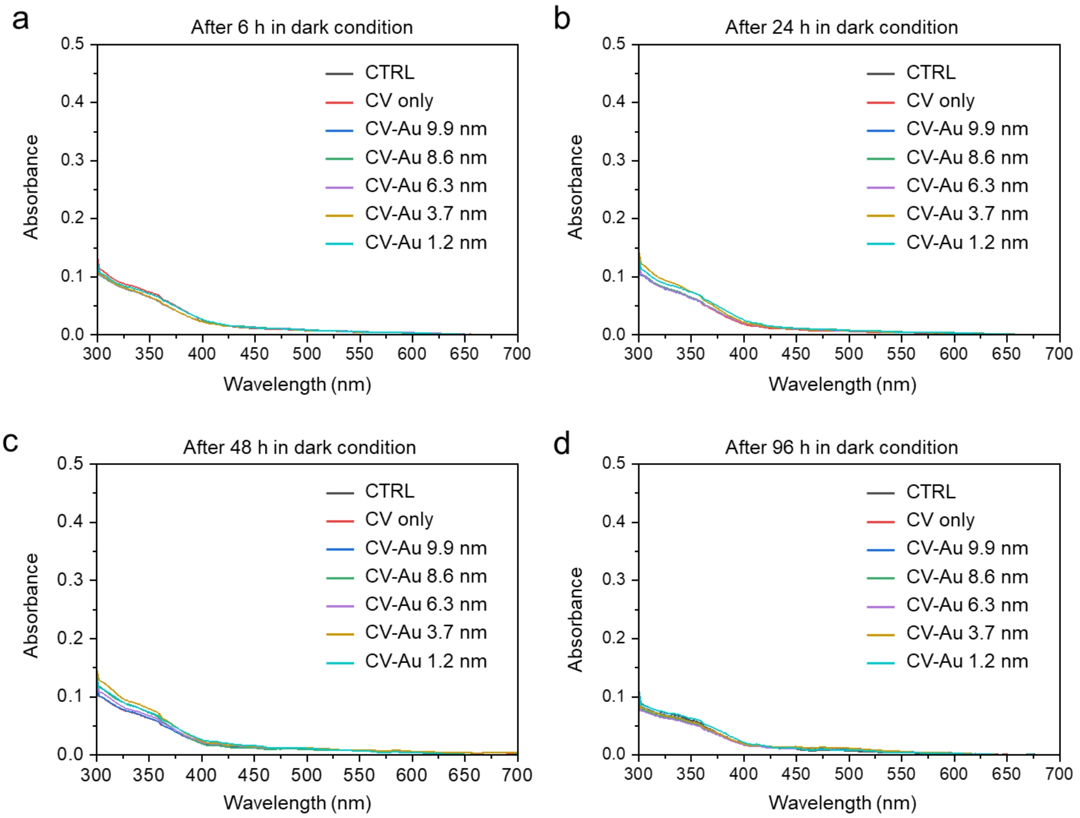

Supporting Figure 16. Change of UV-Vis absorbance spectra by superoxide radicals ($O_2•^{-}$) generated by control (XTT sodium salt solution), crystal violet (CV) only and CV-Au particles treated polymer surfaces after (a) 6, (b) 24, (c) 48 and (d) 96 h incubations in a dark room. XTT sodium salt solution, an $O_2•^{-}$ chemical trap agent, was used. The spectral changes were measured in wavelengths of 300 to 700 nm.

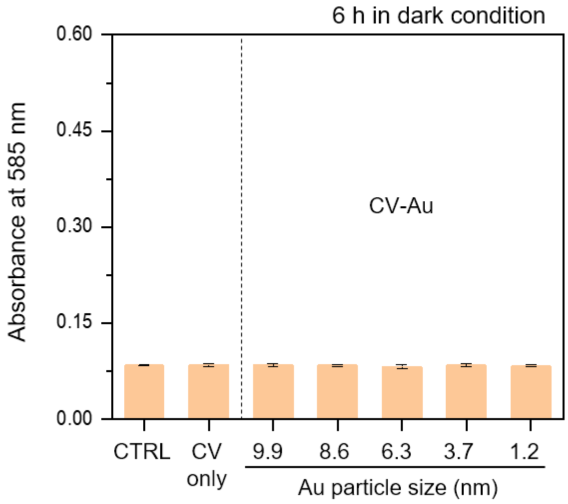

Supporting Figure 17. Light absorbance changes at 595 nm by hydrogen peroxide (H_2O_2) generated by control (Pierce™ quantitative peroxide assay solution), crystal violet (CV) only and CV-Au particles treated polymer surfaces after 6 h incubation in a dark room. Pierce™ quantitative peroxide assay, an H_2O_2 chemical trap agent, was used. The main absorbance change at 595 nm was measured (n=3 independent samples). Data presented as mean ± SD.

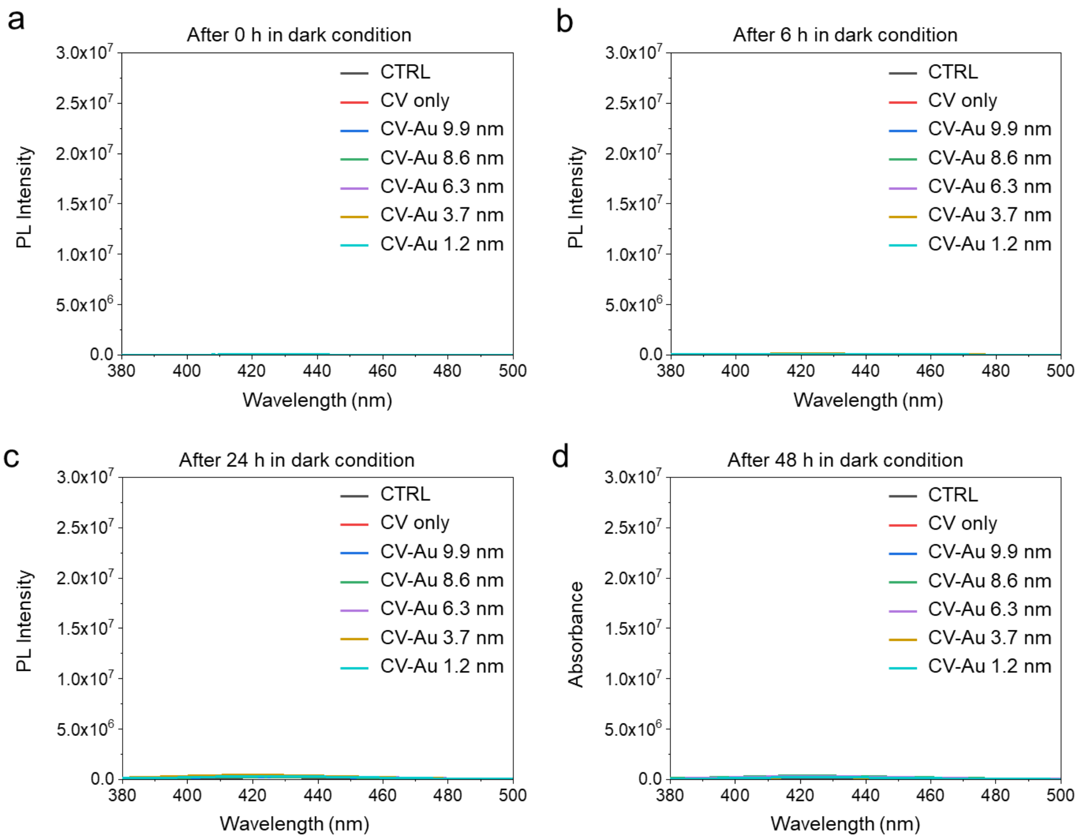

Supporting Figure 18. Change of photoluminescence (PL) spectra by hydroxyl radicals (•OH) generated by control (HTA solution), crystal violet (CV) only and CV-Au particles treated polymer surfaces after (a) 0, (b) 6, (c) 24 and (d) 48 h incubations in a dark room. A 2-hydroxyterephthalic acid (HTA) solution, an •OH chemical trap agent, was used, and the changes of the PL spectra were measured in wavelengths of 380 to 500 nm.

Supporting Table 3 | Recommended guidelines for lighting in healthcare buildings

Location	Illumination (mW cm^{-2})					
	UK [13]	Korea [14]	USA [15]	Germany [16]	Japan [17]	China [18]
Waiting rooms	0.044	0.044	0.029	0.029	-	0.029
Nurse station	0.044	0.146	0.044	0.073	-	0.044
Ward						
Patient reading	0.044	0.029	0.029-0.073	0.029	0.022-0.044	0.015
General care	0.044	0.146	-	0.044	-	-
Operation room						
Overall	0.059-0.073	0.146	0.146-0.293	0.146	0.011-0.022	0.110
Working table	1.46-7.32	-	3.95	2.93-14.64	>2.93	-
Exam room						
Overall	0.044	0.146	0.029-0.073	0.073-0.146	0.029-0.073	0.044
Partial	0.146	-	0.073-0.146	>0.146	0.110-0.220	-
ICU						
Overall	0.004-0.007	0.015	0.015-0.029	0.015-0.044	-	0.044
Partial	0.059	-	0.073-0.146	0.146	-	-
Investigation & treatment						
Overall	0.044	0.073	0.029-0.073	0.073	0.044-0.11	0.073
Partial	0.073	-	0.073-0.146	>0.146	-	-

Lux data in references was converted into values in mW cm^{-2} using equation (8)

Supporting Table 4| Light source, intensity and exposure time, tested bacteria, disinfection activity of visible light active surfaces, which are reported in previous studies

Visible light activated photocatalyst	Light source	Light intensity (mW cm^{-2})	Exposure Time (h)	Tested bacteria	Photodisinfection activity (log reduction)	Ref.
PANI/g-C_3N_4	Xenon lamp with cut off filter	150	0.5	*S. aureus*	1.5	19
CTS/MTiO_2	-	2.4	3	*S. aureus*	3	20
Ag NPs/TiO_2	Fluorescent lamp	0.73	12	*S. aureus*	4	21
3 nm ZnO NPs/crystal violet (CV)	Fluorescent lamp	0.6	1	*S. aureus*	3.4	22
RGO/TiO_2	White LED lamp	9.5	12	*S. epidermidis*	3.6	23
PDMS/Br	Fluorescent lamp	0.59	5	*S. aureus*	3	24
Si-Al/PFOTES-CV	White LED lamp	7.2	3	*S. epidermidis*	4	25
TMB	Warm white light	3.3	6	*S. aureus*	4	26
Ag nanostruct/TiO_2	Incandescent lamp	30	0.17	*S. aureus*	5	27
N doped TiO_2	Incandescent lamp	4.4	0.25	*S. aureus*	0.3	28
PCBPT	White LED light	3.9	6	*S. aureus*	1.8	29
4nm QD/CV	Fluorescent lamp	1	1	*S. aureus*	3.8	30
2nm Au NPs/CVMB	Fluorescent lamp	0.5	3	*S. epidermidis*	3.8	31
CNC-Por	PDT light	60	1	*S. aureus*	6	32
PDMS/CV	Fluorescent lamp	1.5	2	*S. aureus*	3.2	33
$Por^{(+)}$-paper	PDT light	65	0.5	*S. aureus*	5	33
$Bdy^{(2H)}$-paper	PDT light	65	0.5	*S. aureus*	1	34
Au/PCN-224/Cu(II)	Xenon lamp with cut off filter	300	1.3	*S. aureus*	6	35
Au/PCN-224	Xenon lamp with cut off filter	300	1.3	*S. aureus*	4.2	35
PCN-224	Xenon lamp with cut off filter	300	1.3	*S. aureus*	2.1	35
PPIX-ED	Incandescent lamp	8.8	0.5	*S. aureus*	1.5	36
ZnPPIX-ED	Incandescent lamp	5.9	0.5	*S. aureus*	1.4	36

Lux data in references was converted into values in mW cm^{-2} using equation (8)

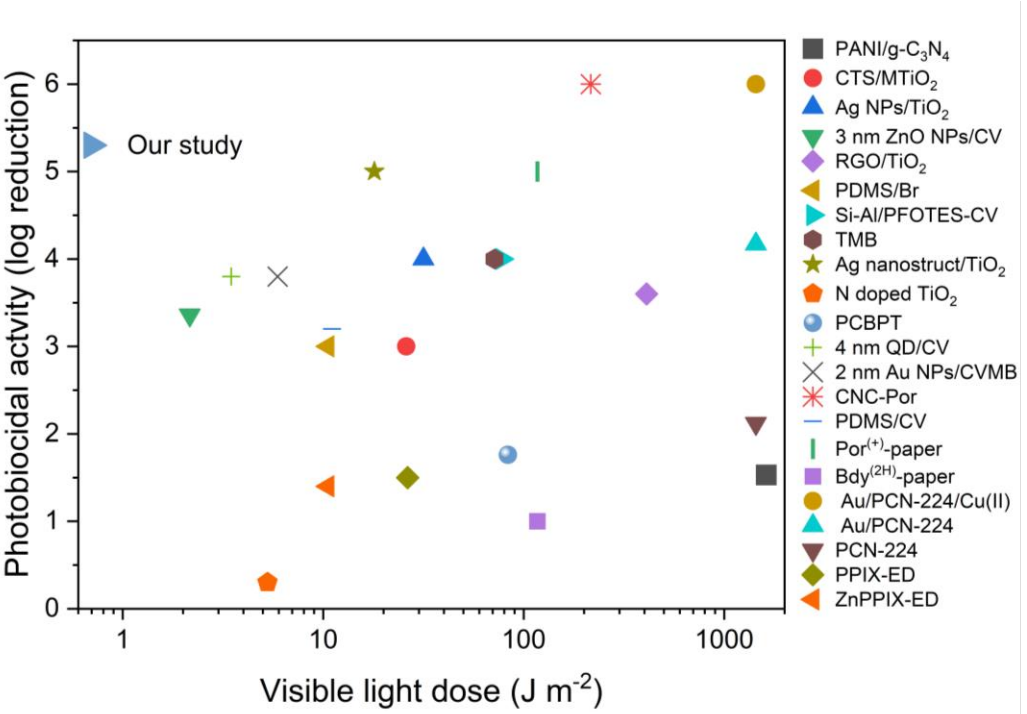

Supporting Figure 19. Comparison of the disinfection activity of various photodisinfection surfaces against *Staphylococcus strains* regarding visible light dose.

References

(1) Hwang, G. B.; Noimark, S.; Page, K.; Sehmi, S.; Macrobert, A. J.; Allan, E.; Parkin, I. P. White Light-Activated Antimicrobial Surfaces: Effect of Nanoparticles Type on Activity. *J. Mater. Chem. B* **2016**, *4*, 2199-2207.

(2) Zhu, M.; Aikens, C. M.; Hollander, F. J.; Schatz, G. C.; Jin, R. Correlating the Crystal Structure of a Thiol-Protected Au_{25} Cluster and Optical Properties. *J. Am. Chem. Soc.* **2008**, *130*, 5883-5885.

(3) Liu, Z.; Zhou, M.; Luo, L.; Wang, Y.; Kahng, E.; Jin, R. Elucidating the Near-Infrared Photoluminescence Mechanism of Homometal and Doped $M_{25}(SR)_{18}$ Nanoclusters. *J. Am. Chem. Soc.* **2023**, *145*, 19969-19981.

(4) Antonello, S.; Perera, N. V.; Ruzzi, M.; Gascon, J. A.; Maran, F. Interplay of Charge State, Lability, and Magnetism in the Molecule-like $Au_{25}(SR)_{18}$ Cluster. *J. Am. Chem. Soc.* **2013**, *135*, 15585-15594.

(5) Juarez-Mosqueda, R.; Mpourmpakis, G. Elucidating the Optical Spectra of $[Au_{25}(SR)_{18}]^{q}$ Nanoclusters. *Phys. Chem. Chem. Phys.* **2019**, *21*, 22272-22282.

(6) Yao, C.; Xu, C. Q.; Park, I. H.; Zhao, M.; Zhu, Z.; Li, J.; Hai, X.; Fang, H.; Zhang, Y.; Macam, G.; et al. Giant Emission Enhancement of Solid-State Gold Nanoclusters by Surface Engineering. *Angew. Chem.* **2020**, *132*, 8347-8353.

(7) Nelson, R. C. Contact Potential Difference between Sensitizing Dye and Substrate. *J. Opt. Soc. Am.* **1956**, *46*, 1016-1019.

(8) Li, G.; Abroshan, H.; Liu, C.; Zhuo, S.; Li, Z.; Xie, Y.; Kim, H. J.; Rosi, N. L.; Jin, R. Tailoring the Electronic and Catalytic Properties of Au_{25} Nanoclusters *via* Ligand Engineering. *ACS Nano* **2016**, *10*, 7998-8005.

(9) Hwang, G. B.; Huang, H.; Wu, G.; Shin, J.; Kafizas, A.; Karu, K.; Toit, H. D.; Alotaibi, A. M.; Mohammad-Hadi, L.; Allan, E.; et al. Photobactericidal Activity Activated by Thiolated Gold Nanoclusters at Low Flux Levels of White Light. *Nat. Commun.* **2020**, *11*, 1207.

(10) Liu, C.; Lin, S.; Pei, Y.; Zeng, X. C. Semiring Chemistry of $Au_{25}(SR)_{18}$: Fragmentation Pathway and Catalytic Active Site. *J. Am. Chem. Soc.* **2013**, *135*, 18067-18079.

(11) Matus, M. F.; Malola, S.; Kinder Bonilla, E.; Barngrover, B. M.; Aikens, C. M.; Hakkinen, H. A Topological Isomer of the $Au_{25}(SR)_{18}^{-}$ Nanocluster. *Chem. Commun.* **2020**, *56*, 8087-8090.

(12) Noimark, S.; Salvadori, E.; Gomez-Bombarelli, R.; MacRobert, A. J.; Parkin, I. P.; Kay, C. W. Comparative Study of Singlet Oxygen Production by Photosensitiser Dyes Encapsulated in Silicone: Towards Rational Design of Anti-Microbial Surfaces. *Phys. Chem. Chem. Phys.* **2016**, *18*, 28101-28109.

(13) British Standards Institution. *Light and Lighting. Lighting of Work Places-Indoor Work Places (BS EN 12464-1)*; London, United Kingdom, 2021.

(14) Korean Standards Association. *Recommended Levels of Illumination (KS A 3011)*; Seoul, South Korea, **1998**.

(15) American Society of Heating and Refrigerating and Air-Conditioning Engineers (ASHRAE). *Energy Standard for Buildings except Low-Rise Residential Buildings (ASHRAE Standard 90.1)*; Atlanta, Georgia, USA, **2010**.

(16) European Committee for Standardization (CEN). *Light and Lighting - Lighting of Work Places - Part 1: Indoor Work Places*; Brussels, Belgium, **2021**.

(17) Japanese Standards Association. *General Rules of Recommended Lighting Levels (JIS Z 9110:2010)*; Tokyo, Japan, **2010**.

(18) China National Standards. *Standard for Lighting Design of Buildings (GB 50034-2013)*; Beijing, China, **2013**.
(19) Hou, J.; Liu, S.; Jiang, X.; Waterhouse, G. I. N.; Zhang, Z.-M.; Yu, L.-m. Polyaniline/Graphite Carbon Nitride Composite Coatings with Outstanding Photo-Induced Anodic Antifouling and Antibacterial Properties under Visible Light. *Prog. Org. Coat.* **2021**, *154*, 106203.
(20) Vélez-Peña, E.; Pérez-Obando, J.; Pais-Ospina, D.; Marín-Silva, D. A.; Pinotti, A.; Cánneva, A.; Donadelli, J. A.; Damonte, L.; Pizzio, L. R.; Osorio-Vargas, P.; et al. Self-Cleaning and Antimicrobial Photo-Induced Properties under Indoor Lighting Irradiation of Chitosan Films Containing Melon/TiO_2 Composites. *Appl. Surf. Sci.* **2020**, *508*, 144895.
(21) Dunnill, C. W.; Page, K.; Aiken, Z. A.; Noimark, S.; Hyett, G.; Kafizas, A.; Pratten, J.; Wilson, M.; Parkin, I. P. Nanoparticulate Silver Coated-Titania Thin Films—Photo-oxidative Destruction of Stearic Ccid under Different Light Sources and Antimicrobial Effects under Hospital Lighting Conditions. *J. Photochem. Photobiol. A* **2011**, *220*, 113-123.
(22) Noimark, S.; Weiner, J.; Noor, N.; Allan, E.; Williams, C. K.; Shaffer, M. S. P.; Parkin, I. P. Dual-Mechanism Antimicrobial Polymer-ZnO Nanoparticle and Crystal Violet-Encapsulated Silicone. *Adv. Funct. Mater.* **2015**, *25*, 1367-1373.
(23) Jeong, S. B.; Heo, K. J.; Yoo, J. H.; Kang, D. G.; Santoni, L.; Knapp, C. E.; Kafizas, A.; Carmalt, C. J.; Parkin, I. P.; Shin, J. H.; et al. Photobiocidal Activity of TiO_2/UHMWPE Composite Activated by Reduced Graphene Oxide under White Light. *Nano. Lett.* **2024**, *24*, 9155-9162.
(24) Peveler, W. J.; Noimark, S.; Al-Azawi, H.; Hwang, G. B.; Crick, C. R.; Allan, E.; Edel, J. B.; Ivanov, A. P.; MacRobert, A. J.; Parkin, I. P. Covalently Attached Antimicrobial Surfaces using BODIPY: Improving Efficiency and Effectiveness. *ACS Appl. Mater. Interfaces* **2018**, *10*, 98-104.
(25) Jeong, S. B.; Lee, D. U.; Lee, B. J.; Heo, K. J.; Kim, D. W.; Hwang, G. B.; MacRobert, A. J.; Shin, J. H.; Ko, H. S.; Park, S. K.; et al. Photobiocidal-Triboelectric Nanolayer Coating of Photosensitizer/Silica-Alumina for Reusable and Visible-Light-Driven Antibacterial/Antiviral Air Filters. *Chem. Eng. J.* **2022**, *440*, 135830.
(26) Merkes, J. M.; Mukherjee, T.; Chiera, S.; Laukkanen, O. V.; Bruenke, J.; Brust, T.; Tessarolo, F.; Kiessling, F.; Rueping, M.; Banala, S. Antimicrobial Personal Protection Clothing: Development of Visible Light Activated Antimicrobial Coatings for Nonwoven Polypropylene Fibers. *Adv. Mater. Interfaces* **2023**, *11*, 2300601.
(27) Wong, M. S.; Sun, D. S.; Chang, H. H. Bactericidal Performance of Visible-Light Responsive Titania Photocatalyst with Silver Nanostructures. *PLoS One* **2010**, *5*, e10394.
(28) Wong, M. S.; Chu, W. C.; Sun, D. S.; Huang, H. S.; Chen, J. H.; Tsai, P. J.; Lin, N. T.; Yu, M. S.; Hsu, S. F.; Wang, S. L.; et al. Visible-Light-Induced Bactericidal Activity of a Nitrogen-Doped Titanium Photocatalyst against Human Pathogens. *Appl. Environ. Microbiol.* **2006**, *72*, 6111-6116.
(29) Musolino, S. F.; Shatila, F.; Tieman, G. M. O.; Masarsky, A. C.; Thibodeau, M. C.; Wulff, J. E.; Buckley, H. L. Light-Induced Anti-Bacterial Effect against *Staphylococcus Aureus* of Porphyrin Covalently Bonded to a Polyethylene Terephthalate Surface. *ACS Omega* **2022**, *7*, 29517-29525.
(30) Owusu, E. G. A.; MacRobert, A. J.; Naasani, I.; Parkin, I. P.; Allan, E.; Yaghini, E. Photoactivable Polymers Embedded with Cadmium-Free Quantum Dots and Crystal

violet: Efficient Bactericidal Activity against Clinical Strains of Antibiotic-Resistant Bacteria. *ACS Appl. Mater. Interfaces* **2019**, *11*, 12367-12378.
(31) Noimark, S.; Allan, E.; Parkin, I. P. Light-Activated Antimicrobial Surfaces with Enhanced Efficacy Induced by a Dark-Activated Mechanism. *Chem. Sci.* **2014**, *5*, 2216-2223.
(32) Feese, E.; Sadeghifar, H.; Gracz, H. S.; Argyropoulos, D. S.; Ghiladi, R. A. Photobactericidal Porphyrin-Cellulose Nanocrystals: Synthesis, Characterization, and Antimicrobial Properties. *Biomacromolecules* **2011**, *12*, 3528-3539.
(33) Ozkan, E.; Allan, E.; Parkin, I. P. The Antibacterial Properties of Light-Activated Polydimethylsiloxane Containing Crystal Violet. *RSC Adv.* **2014**, *4*, 51711-51715.
(34) Carpenter, B. L.; Scholle, F.; Sadeghifar, H.; Francis, A. J.; Boltersdorf, J.; Weare, W. W.; Argyropoulos, D. S.; Maggard, P. A.; Ghiladi, R. A. Synthesis, Characterization, and Antimicrobial Efficacy of Photomicrobicidal Cellulose Paper. *Biomacromolecules* **2015**, *16*, 2482-2492.
(35) Xue, J.; Zhang, J.; Yuan, M.; Lv, Y.; Chen, Z.; Wang, M. Visible-Light-Driven Au/PCN-224/Cu(II) Modified Fabric with Enhanced Photocatalytic Antibacterial and Degradation Activity and Mechanism Insight. *Sep. Purif. Technol.* **2024**, *333*, 125863.
(36) Bozja, J.; Sherrill, J.; Michielsen, S.; Stojiljkovic, I. Porphyrin-based, Light-Activated Antimicrobial Materials. *J. Polym. Sci. Part A: Polym. Chem.* **2003**, *41*, 2297-2303.
